# Short-term 3D culture systems of various complexity for treatment optimization of colorectal carcinoma

**DOI:** 10.1038/s41598-019-42836-0

**Published:** 2019-05-08

**Authors:** Marloes Zoetemelk, Magdalena Rausch, Didier J. Colin, Olivier Dormond, Patrycja Nowak-Sliwinska

**Affiliations:** 1 Molecular Pharmacology Group, School of Pharmaceutical Sciences, University of Geneva, University of Lausanne, 1211 Geneva 4, Switzerland; 2 Translational Research Center in Oncohaematology, 1211 Geneva 4, Switzerland; 3 Centre for BioMedical Imaging (CIBM), University Hospitals and University of Geneva, 1211 Geneva 4, Switzerland; 4 Department of Visceral Surgery, Lausanne University Hospital, Lausanne, Switzerland

**Keywords:** Targeted therapies, Tumour heterogeneity

## Abstract

Three-dimensional (3D) cultures have the potential to increase the predictive value of pre-clinical drug research and bridge the gap towards anticipating clinical outcome of proposed treatments. However, their implementation in more advanced drug-discovery programs is still in its infancy due to the lack of reproducibility and low time- and cost effectiveness. HCT116, SW620 and DLD1 cells, cell lines with distinct mutations, grade and origin, were co-cultured with fibroblasts and endothelial cells (EC) in 3D spheroids. Clinically relevant drugs, i.e. 5-fluorouracil (5−FU), regorafenib and erlotinib, were administered individually to in CRC cell cultures. In this study, we established a robust, low-cost and reproducible short-term 3D culture system addressing the various complexities of the colorectal carcinoma (CRC) microenvironment. We observed a dose-dependent increase of erlotinib sensitivity in 3D (co-)cultures compared to 2D cultures. Furthermore, we compared the drug combination efficacy and drug-drug interactions administered in 2D, 3D and 3D co-cultures. We observed that synergistic/additive drug-drug interactions for drug combinations administered at low doses shifted towards additive and antagonistic when applied at higher doses in metastatic CRC cells. The addition of fibroblasts at various ratios and EC increased the resistance to some drug combinations in SW620 and DLD1 cells, but not in HCT116. Retreatment of SW620 3D co-cultures with a low-dose 3-drug combination was as active (88% inhibition, relative to control) as 5-FU treatment at high dose (100 μM). Moreover, 3D and 3D co-cultures responded variably to the drug combination treatments, and also signalling pathways were differently regulated, probably due to the influence of fibroblasts and ECs on cancer cells. The short-term 3D co-culture system developed here is a powerful platform for screening (combination) therapies. Understanding of signalling in 3D co-cultures versus 3D cultures and the responses in the 3D models upon drug treatment might be beneficial for designing anti-cancer therapies.

## Introduction

Colorectal cancer (CRC) remains a leading cause of cancer-related deaths despite several improvements in early detection and treatment options; it has an approximated yearly incidence of 1.4 million new cases and seven hundred thousand deaths globally^1,2^. First-line treatment at early stages includes resection of the primary tumor, which can be curative for patients with local disease. Patients with invasive tumors and risk of relapse can benefit from adjuvant chemotherapy consisting of a combination of 5-fluorouracil (5-FU) with other chemotherapeutics^3^. However, 40–50% of diagnosed patients will eventually develop metastatic colorectal carcinoma (mCRC) and resistance to the administered chemotherapies. Addition of targeted therapies with molecules targeting vascular endothelial growth factor (VEGF), such as bevacizumab and Zaltrap^®^, VEGF receptor 2 (VEGFR2, i.e. regorafenib and ramucirumab), as well as the epidermal growth factor receptor (EGFR, i.e. erlotinib, cetuximab and panitumumab) resulted in improved overall survival of mCRC patients^4^. The five-year survival rates for patients diagnosed with early stage of localized or regional CRC is currently between 70–90%. However, this result drops to only 14% for patients with late stage metastatic disease^2^, underlining the need for improved treatment options. Advances in drug development have resulted in a record number of new FDA approvals in 2017 with 18 new drugs and 13 repurposed drugs^5^. The current success rate of approval for anti-cancer compounds entering clinical trials is still only 4%, underscoring the inefficiency in translating new treatment options to clinical success^6,7^. Therefore, improving pre-clinical or *in vitro* models as predictors of drug efficacy and safety^8,9^ can help to improve drug development.

*In vitro* drug screening is often performed using 2-dimensional (2D) homotypic tumor cell culture systems. Three-dimensional (3D) *in vitro* cell culture models, consisting of co-culture systems of tumor cells and stromal cell types, can increase the predictive value of pre-clinical *in vitro* drug discovery and development by closely recapitulating the disease model and the response to anti-cancer treatments^10–12^. 3D cultures can more realistically mimic the clinical presentation and response to treatment of the tumor and have the potential to reduce the gap between *in vitro* drug development and further validation and translation^13–16^. In addition, 3D culture systems are extremely well suited for screening of personalized strategies. As in tumors, the growth of tumor cells in 3D spheroid cultures involves the presence of oxygen- and nutrient gradients^15,17^. As a result, cell proliferation and cell death rates vary within the spheroid, affecting the overall growth and response of the spheroid to administered treatments^11,12^. Furthermore, it is known that stromal cells integrated in 3D cultures can affect the response of tumor cells to treatment^18,19^. Incorporation of components of the tumor microenvironment and interacting cell types may improve the relevance of this model in drug screening^18,19^. In CRC, fibroblasts are major players contributing to tumor development, progression, induction of metastasis, tumor angiogenesis and suppression of the immune response, through secretion of a wide range of molecules that mediate tumor-fibroblast cross talk^20–22^.

Previously reported CRC 3D co-cultures include spheroids mimicking tumor angiogenesis^23^ and microfluidic systems enabling study of the metastasis and interactions with immune cells and fibroblasts^24–26^. However, These systems are expensive, have a low-throughput setup, are highly variable and incompatible with straightforward analysis methods. They are therefore not suitable for large-scale drug screening. However, polystyrene-coated low-attachment round-bottom plates can be used to reproducibly form single spheroids with easy access for analysis. The cells can be seeded in the presence of low percentages of basement membrane (BM) to promote spheroid formation without increasing the viscosity or gelation/polymerization of the culture medium^27,28^.

The aim of our study was to design a robust and reproducible short-term 3D culture system including multiple cellular components of the CRC microenvironment, compatible with optimization of (personalized) drug combinations. We compared drug dose-response curves of three clinically relevant drugs and their combination efficacy in 2D, 3D and 3D co-cultures by measuring cell viability, based on metabolic activity. Optimized culture systems were used in a search for cell-type (patient-specific) drug combinations. One of the important results of this study were the morphological and physiological changes observed with the integration of fibroblasts and endothelial cells to the 3D cultures, and the variations in drug combination efficacy between the various cell types and culture systems.

## Methods

### Drugs

Erlotinib HCL (E-4007, LC laboratories), 5-fluorouracil (F6627, Sigma-Aldrich) and regorafenib (S1178, Selleck Chemicals) were dissolved in sterile DMSO (Sigma-Aldrich) at concentrations of 15 mg/mL, 10 mg/mL and 20 mg/mL, respectively. Aliquots were stored at −80 °C and thawn prior to each experiment for one-time use. A maximal concentration of 0.1% DMSO was used as control.

### Cell lines and culture conditions

Human CRC and CCD841 CoN cell lines were obtained from ATCC or Public Health England with a corresponding authentication certificate. Human immortalized endothelial cells ECRF24 cells were generously donated by Prof. AW Griffioen (Angiogenesis Laboratory, UMC Amsterdam). The cells were cultivated at 37 °C in a humidified atmosphere with 5% CO_2_ in culture medium supplemented with 10% foetal bovine serum (FCS) (S1810-500, Biowest) and 1% penicillin/streptomycin (4-01F00-H, BioConcept). HCT116, SW620, HT29, SW48, LS174T and Caco2 were cultured in DMEM Glutamax medium (31966-021, Gibco), DLD1 in RPMI-1640 Glutamax medium (61870-010, Gibco), ECRF24 in DMEM/RPMI 1:1 on a 0.2% gelatin coated surface (G1393-100ML, Sigma), CCD841 and CCD18co in EMEM medium (M2279-500ML, Sigma) additionally supplemented with 2 mM L-Glutamin (25030024, Gibco). Cells were monitored for *mycoplasma* contamination using the MycoAlert kit (LT07-218, Lonza).

### Establishing 2D, 3D and 3D co-cultures

2D cell cultures were established in flat-bottom 96-well plates (353072, Falcon) and seeded 2500, 2500 and 5000 cells/well for HCT116, DLD1 and SW620, respectively. 3D cultures were established by seeding cells at optimized densities between 1000–1500 cells/well in 96-well U-bottom low attachment plates (650970, Greiner) in their respective culture media. 3D co-cultures were obtained by mixing the CRC cells with 30%, 50% or 70% normal human fibroblasts (CCD18co) to 1000 cells/well and the addition of 5% ECRF24. The cell culture medium of the 3D co-cultures was a mixture of DMEM, RPMI and EMEM (Supplementary Fig. S4) supplemented with 2.5% Matrigel^TM^ (354254, Corning). Spheroid growth was measured using (i) spheroid size and (ii) metabolic activity as an indicator of cell viability.

### Monitoring spheroid growth and circularity

Spheroid size was measured with the BioTek Cytation 3 imaging reader with corresponding Gen5 Image software version 3.04. A Z-stack of each spheroid was obtained in brightfield with a 4x objective and Z-projection was performed using focus stacking settings. Spheroid size and circularity was calculated using the cellular analysis feature of the software using: dark objects on a bright background, do not split touching objects, threshold 15.000 RFU (Relative Fluorescence Unit).

### Metabolic activity assay

Metabolic activity assays were performed on HCT116, DLD1 and SW620 cells grown in 2D, 3D and 3D co-cultures. Post-treatment cell viability was measured using the luminescent-based cell metabolic activity assay CellTiter-Glo^®^ (G7572, Promega) for 2D cultures and 3D-CellTiter-Glo^®^ (G9683, Promega) for 3D and 3D co-cultures, according to the manufacturer’s instructions. The intensity of the luminescence signal was detected with the BioTek Cytation 3 imaging reader with corresponding Gen5 Image software version 3.04, using standard settings.

### Treatment

Single drugs or pre-mixed drug combinations were incubated for 72 hours for 2D cultures at day 1 post-seeding and for 3D and 3D co-cultures at day 1, 2 or 4 post-seeding. For retreatment of the cells, the cells were re-incubated for an additional 48 hours. Drugs were administered at low doses (LD, corresponding to the EC_20_ for each cell type) or at the maximal plasma concentration (MPC). The MPC is converted from the area under the curve (AUC)_0–24_ after clinical treatment with standard or maximally tolerated doses into the average plasma concentration from 0 to 24 hours, i.e. 50.26 mg h l^−1^ for regorafenib, 15.2 µg*h/mL for erlotinib and 20–30 mg*h/mL for 5-FU (see Supplementary Table S3). The cell culture medium and solvent controls were included and used for calculating drug efficacy relative to 100% control.

### Calculation of the combination index

CompuSyn software (based on the Loewe additivity model) was used to calculate the combination index of the drug combinations tested^29^. The combination index generated was categorized as synergistic (<0.8), additive (0.8–1.0) or antagonistic (>1.0).

### LIVE/DEAD cell double staining

Live-dead staining was performed on the 3D and 3D co-cultures using calcein (17783-1MG, Sigma) and ethidium homodimer (EtHD) (46043-1MG-F, Sigma). Spheroids were washed once with PBS and stained for 45 min with a mixture of 4 µM calcein, 10 µM EtHD and 5 ug/mL DAPI, and fluorescent imaging was performed.

### CellTracker Staining

Prior to cell harvesting, CRC cells, fibroblasts and endothelial cells were washed with PBS and incubated in serum free medium for 20 minutes supplemented with 40 µM blue CMAC, 5 µM Green CMFDA or 10 µM Red CMTPX (C2110, C7025 and C34552, Life Technologies), respectively. Afterwards, 3D co-cultures were established and fluorescent imaging was performed.

### Immunohistochemistry

HCT116 homotypic 3D, 3D co-cultures were paraffin embedded. Cross-sections were characterized with Hematoxylin & Eosin (H&E) or immunohistochemical staining for the expression of cleaved caspase 3 (ClCasp3), a marker for apoptosis, and the proliferation marker (Ki67), see Supplementary Material.

### Fluorescence imaging

Fluorescent images were obtained using the BioTek Cytation 3 imaging reader with corresponding Gen5 Image software version 3.04. Fluorescent signal images were obtained for LIVE/DEAD stained cells, CellTracker incubated cells and IHC stained cells using the DAPI, GFP and Texas Red filter cubes with the 4x and 10x objectives. A Z-stack of each spheroid was taken and a Z-projection was obtained using focus stacking. For kinetic movies of spheroid formation, temperature control was set to 37 °C and an atmosphere of 5% CO_2_ prior to imaging. Kinetic movies were taken over a period of 12-18h with 30 minutes interval.

### Western Blot

3D and 3D co-cultures were grown in 96-well low-attachment plates and treated as indicated. Spheroids were washed twice with ice-cold PBS, disassociated with Accumax solution, washed again and lysed in RIPA buffer containing protease inhibitor cocktail (Roche, Basel, Switzerland) and PhosSTOP (Roche, Basel, Switzerland). Protein concentrations were evaluated in lysates using Bradford assay (Thermo Fischer Scientific, Waltham, MA, USA). Fifteen µg of proteins per condition was separated on 4–12% polyacrylamide gels (Invitrogen, Waltham, MA, USA) and transferred to a polyvinylidene difluoride membrane. Odyssey blocking buffer (LI-COR Biosciences, Lincoln, NE, USA) was used to block membranes and following incubation with primary and infrared secondary antibodies, bands from immunoreactive proteins (see Supplemental Table 2) were visualized by an Odyssey infrared imaging system at 700 nm for α-mouse and at 800 nm for α-rabbit stained proteins. Of note, following staining for fibronectin, a second staining was performed for laminin on the same blots. Analysis was performed using Image Studio^TM^ Lite software. Images were obtained with the Licor Odyssey CLx scanner at one default exposure setting. To distinguish neighbouring bands brightness was adjusted in Image Studio^TM^ Lite per blot row for sufficient band quantification.

### Statistical analysis

All data is presented as the mean of minimally two independent experiments with corresponding error bars of standard deviation (SD) or the standard error of the mean (SEM), as indicated in the figure legends. Data analysis was performed using Graphpad Prism version 7.02 using the one-way or two-way ANOVA test with post-hoc multiple comparison tests or a student’s t-test, as specified in the figure legends. Statistical significance was indicated with *p < 0.05, **p < 0.01 and ***p < 0.001.

The coefficient of variation (CV%) were calculated from the means of at least 3 independent experiments (in triplicate) according to standard formula. For the calculation of CV the names of the drugs were coded in order to guaranty unbiased operation. Standard acceptance criteria are CV% <20.

## Results

### CRC 3D culture optimization and characterization

**Figure 1:**
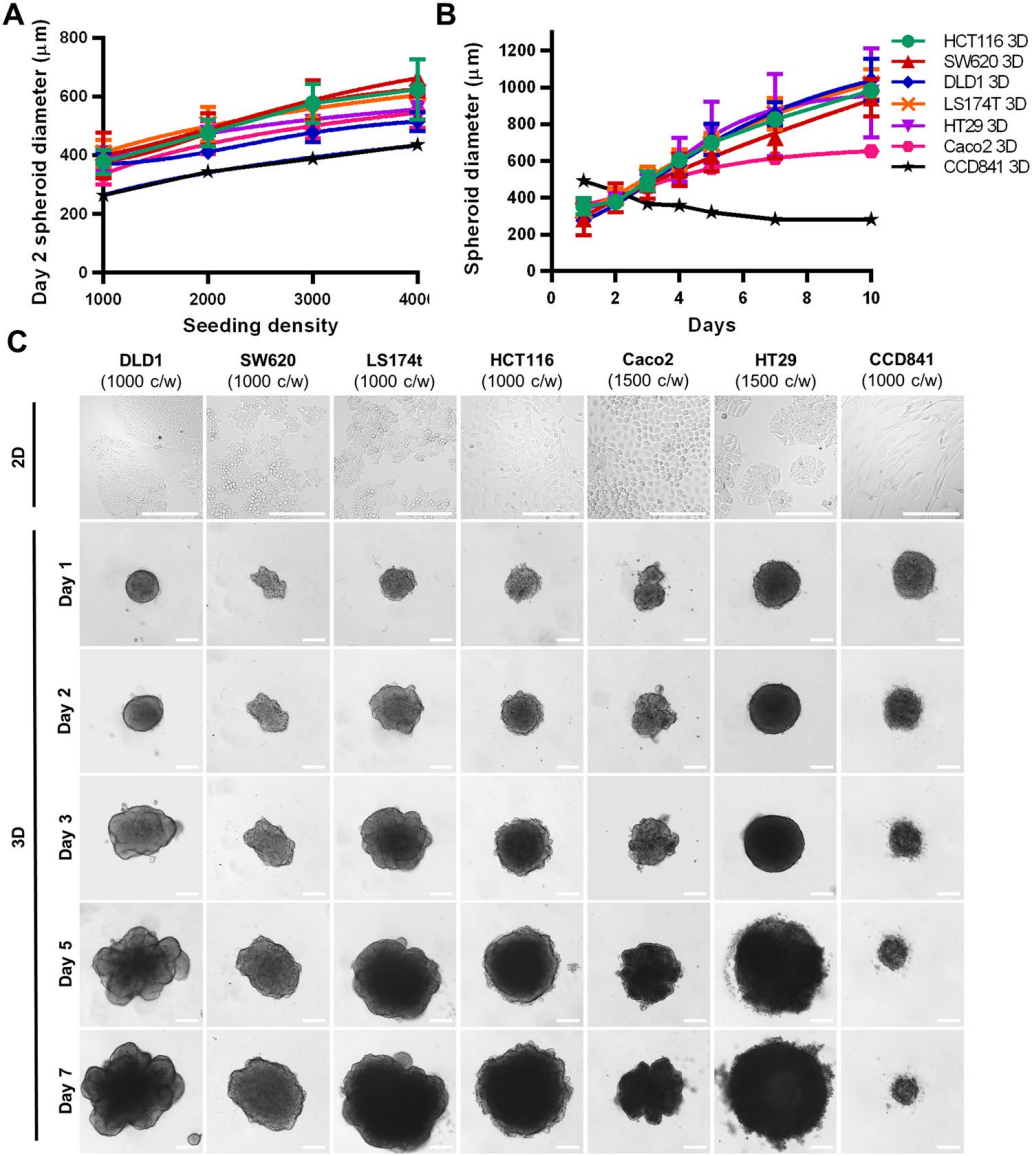
CRC spheroid kinetics and morphology. (**A**) Spheroid diameter of cells 48 hours post-seeding of 1000–4000 cells/well. (**B**) Growth kinetics represented in spheroid diameter over 7 days at optimized seeding densities of 1000 cells/well for DLD1, SW620, LS174T, HCT116 and HT29, and 1500 cells/well for Caco2. Error bars represent the standard deviation, n = 3–15. (**C**) Representative images of the CRC spheroids and the healthy colon epithelial cell line (CCD841 CoN) grown at the optimal seeding densities between day 1 and 7. Scale bar represent 200 µm.

Seven human CRC cell lines varying in genetic background, stage and morphology (see Table 1), were tested for their ability to form reproducible 3D spheroids *in vitro*. In order to enable spheroid formation we used 96-well round-bottom low attachment plates to prevent adhesion to the well bottom and to facilitate cell-cell contact and rapid formation of round or spherical structures within a day. The cell culture medium was supplemented with 2.5% basement membrane (BM) extract to provide additional extracellular matrix components (Fig. 1). It was observed that the addition of BM supported spheroid formation and prevented the formation of non-spherical loose cell aggregates (Supplemental Fig. S1). Of note, methylcellulose, also reported to promote spheroid formation, was investigated for comparison, but did not result in improved spheroid formation in comparison to Matrigel^TM^ in our hands (Supplementary Figs S6–S8).

**Table 1:** Panel of CRC cell lines used in 3D cultures.

Cell line	Origin	Duke’s type	MSI/CIN status	Mutations/deregulations	Doubling time in 2D (h)	Ref
DLD1	Metastatic	C	MSI	APC^l1417fs,R2166^, KRAS^G13D^, PIK3CA^E545K;D549N^, TP53^S241F^	20	^68– 71^
SW620	Metastatic	C	MSS; CIN^pos46^	APC^Q1338^, KRAS^G12V^, TP53^R273H;P309S^	31	^70– 73^
HCT116	Primary	A	MSI	KRAS^G13D^, PIK3CA^H1047R^,	20–24	^70, 71, 73, 74^
LS174T	Primary	C	MSI	KRAS^G12D^, PIK3CA^H1047R^,	32	^70, 71, 75^
HT29	Primary	C	MSS	APC^E853;T1556fs^, BRAF^V600E^, PIK3CA^P449T^, TP53^R273H^,	20–24	^70, 76^
Caco2	Primary	B	MSS; CIN^pos48^	TP53	20–24	^70, 77^
SW48	Primary	C	MSI	EGFR^G719S^	31	^70, 72, 78^

MSI: micro-satellite instability; MSS: micro-satellite stability; CIN: chromosomal instability pathway.

Seeding density was optimized to 1000–1500 cells/well for all CRC cells types in order to obtain spheroids with a diameter of 350–400 µm on day 2 post seeding (Fig. 1A,B, Supplementary Fig. S2). All CRC cell types could be maintained for ten days (Fig. 1B). As measured by diameter, the CRC homotypic spheroids had similar growth kinetics reaching 1100 µm at day 10, whereas Caco2 3D cultures grew at a somewhat slower rate (Fig. 1B). Most of the CRC spheroids formed compact round or spherical structures by day 1, which increased in size over time and presented a remodeled periphery. Of note, SW48 cells did not form 3D structures but irregularly shaped cell aggregates in all conditions (Supplementary Fig. S1). The normal colon epithelial cells (CCD841 CoN) were able to form spheroids, but in contrast to the CRC spheroids, the compactness increased over time leading to a decrease in spheroid size (Fig. 1B,C). The phenotypic features observed for all CRC spheroid types are summarized in Supplementary Table S1.

### Treatment optimization of CRC 3D cultures

Three CRC cell types, characterized with distinct mutations, tumor grade and morphology in 3D cultures, were selected for further investigation, i.e. DLD1, SW620 (both of metastatic origin, high tumor grade), and HCT116 cells (of primary tumor origin, low grade). Spheroids of these cells were exposed for 72 hours to pyrimidine analog 5-fluorouracil (5-FU), the EGFR targeting tyrosine kinase inhibitor (TKI) erlotinib or the multi-TKI regorafenib targeting mainly VEGFR-2, -3, Ret, Kit, PDGFR and Raf.

In the first step, dose-response curves were established for each cell line and for each drug a comparison was made using two parameters for treatment efficacy: (i) spheroid size (Supplementary Fig. S3A) and (ii) metabolic activity of the cells, measured in ATP levels (Supplementary Fig. S3B). Spheroid size plateaued at 20–40% inhibition versus control (CTRL, i.e. 0.1% DMSO for all conditions) for each drug and each cell line. For metabolic activity a dose-dependent decline could be observed for each drug and each cell type. Near complete inhibition of metabolic activity was obtained in the 3D cultures after administration of regorafenib at a high dose (30 μM). Similarly, the efficacy of erlotinib and 5-FU was significantly higher using this readout as compared to spheroid size (Supplementary Fig. S3B).

Next, we compared activity of the treatments with each drug administered for 72 hours at two schedules, i.e. (i) starting 24 hours after spheroids formation was started (Supplementary Fig. S3A,B, open symbols), or (ii) starting 48 hours after spheroid formation was started, when more compact 3D cultures were established (Supplementary Fig. S3A,B, closed symbols). The use of spheroid size as a readout in our short-term cultures did not reveal any significant differences between the treatment schedules (Supplementary Fig. S3A). However, at analyzing cell metabolic activity we noticed that 3D cultures treated with schedule (ii) were significantly less sensitive to regorafenib at 30 μM in DLD1 cells and to 5-FU in SW620 3D cultures at high doses (Supplementary Fig. S3B). Therefore, for further experiments we selected cell metabolic activity as a sensitive and reliable readout and schedule (ii) with 72 hours treatment incubation starting from day two for all further experiments.

### Cell type specific variations in drug sensitivity in 2D and 3D cultures

**Figure 2:**
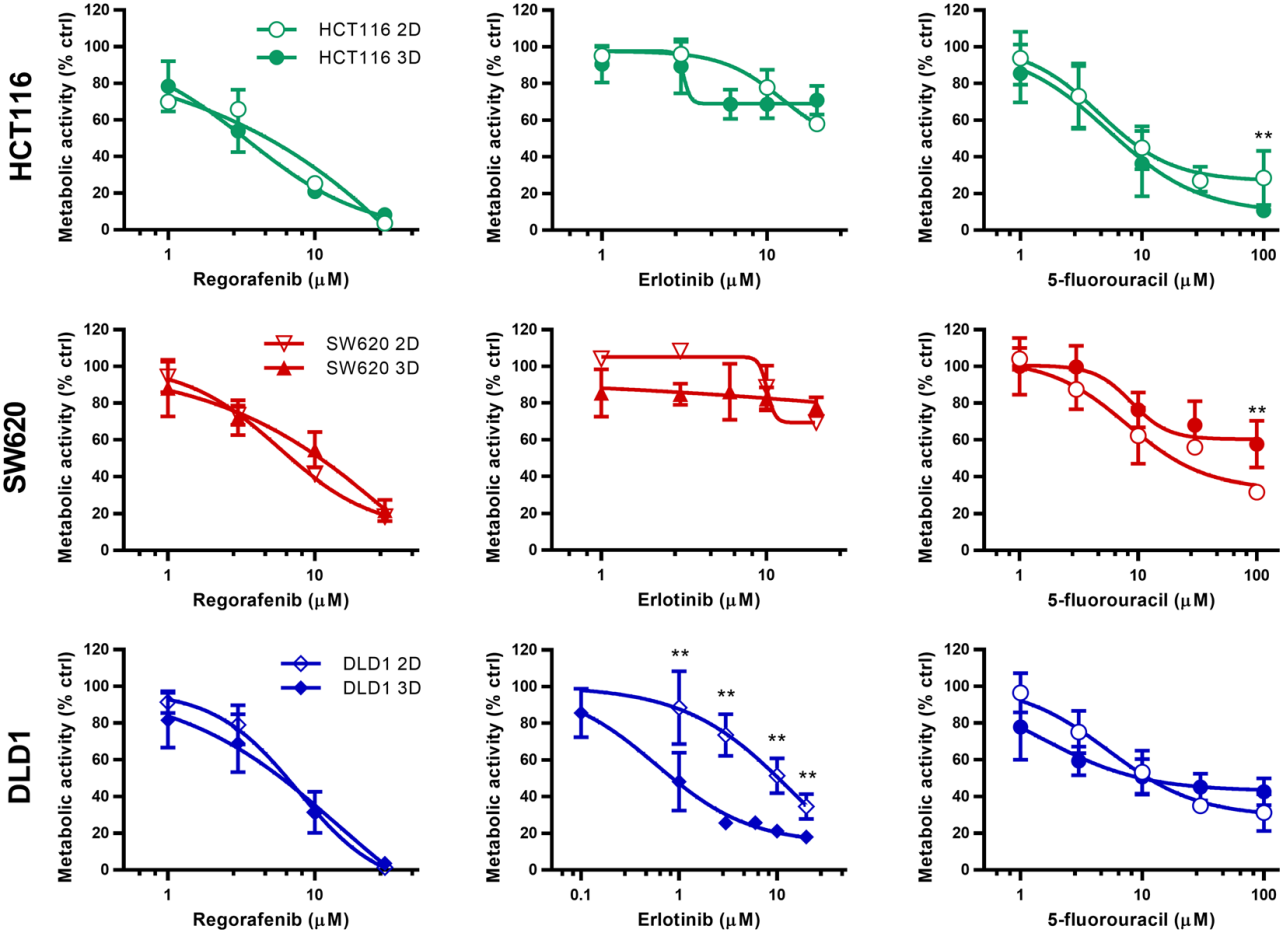
Dose response curves for regorafenib, erlotinib and 5-fluorouracil in 2D and 3D cultures. Metabolic activity dose-response curves for HCT116, SW620 and DLD1 2D and 3D cultures after 72 hours treatment with regorafenib, erlotinib or 5-fluorouracil. Error bars represent the standard deviation for n = 3–15. Significances of *p < 0.05 and **p < 0.01 represent the difference for each drug dose between 2D and 3D cultures, determined with the unpaired multiple T-test.

In order to determine if a change in drug sensitivity occurs when cells are cultured in 3D, we compared single drug activity dose response curves of regorafenib, erlotinib and 5-FU between the 2D and 3D homotypic cultures for each cell line (Fig. 2). Response to regorafenib was similar for all cell lines in both 2D and 3D systems. Interestingly, a difference between the 2D and 3D cultures was observed for 5-FU and erlotinib. While 5-FU efficacy was significantly reduced in SW620 and HCT116 3D cultures, significantly increased sensitivity to erlotinib treatment was observed in DLD1 3D cultures. Based on the dose response curves the effective concentrations inhibiting 20% (low dose, LD) and 50% (EC_50_) of the cells were established (Supplementary Table S3). The clinically relevant drug maximal plasma concentrations (MPC) were calculated based on literature (Supplementary Table S3). In most cases, the EC_50_ values were similar in the 2D and 3D cultures. However, the EC_50_ for erlotinib was 2-fold and 25-fold lower in the 3D homotypic cultures for HCT116 and DLD1 cells, respectively.

### CRC 3D co-cultures with fibroblasts and endothelial cells have unique characteristics

**Figure 3:**
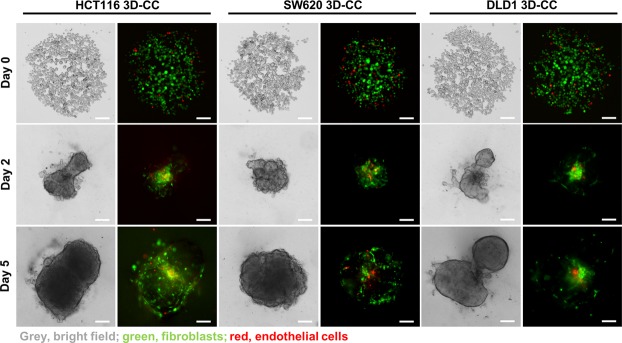
Intra-spheroid localization of CRC cells, fibroblasts and endothelial cells over time. Representative images of DLD1, HCT116 and SW620 3D-CC seeded at 1000 cells/well recorded on day 0, 2 and 5. Cultures consist of tumor cells (bright field, grey), CCD18co healthy colon fibroblasts (green) in ratio 1:1 and 5% endothelial ECRF24 cells (red). Scalebar represents 200 µm.

CRC progression is in part enabled by the tumor microenvironment, which contains abundant stromal cells including tumor-associated fibroblasts and activated endothelial cells. Therefore, we created 3D co-cultures (3D-CC) consisting of CRC cells (DLD1, SW620 or HCT116) with normal human colon fibroblasts (CCD18co) in a clinically relevant ratio of 1:1 and with 5% of human immortalized endothelial cells (ECRF24)^30^. The co-cultured cells were stained with CellTracker^®^ in order to follow the intra-spheroid localization of the cells in time (Fig. 3 and Supplementary Movies S1–S3).

Directly after seeding (day 0) we observed the cells forming spheroids. At day 2, the 3D-CC of DLD1 and HCT116 spheroids presented heterogeneous shapes, typically with two spherical growths connected in the center through mostly fibroblasts and endothelial cells. In contrast, the SW620 3D-CC created a circular spheroid with the fibroblasts and endothelial cells distributed throughout the spheroid. Similar morphologies were observed at day 5 of experiments.

Decreased size of the 3D-CCs compared to the 3D homotypic cultures was observed for each cell type, with reductions of 18%, 21% and 23% for HCT116, SW620 and DLD1, respectively. Increased heterogeneity in DLD1 shapes was confirmed with a decrease in circularity, which determines how close to a spherical geometry shape they are, from 0.610 ± 0.065 in the DLD1 3D homotypic cultures to 0.529 ± 0.04 in the 3D-CCs, significantly lower than the circularity of HCT116 (0.0759 ± 0.032) and SW620 (0.809 ± 0.015) 3D-CCs. Overall, for each of the CRC cell types the 3D-CC had distinct characteristics and more heterogeneous morphometric features as compared to the same cells grown in simple 3D cultures.

### Drug sensitivity is culture system and cell line dependent

In the next step, we compared the activity of various drug combinations in all cell culture systems, i.e. 2D, 3D homotypic and 3D-CC (heterotypic), see Supplementary Table S4. Drug interactions were determined based on the results of the drug combinations with a combination index (CI), which was calculated with Compusyn^®^ software categorizing combinations as synergistic (<0.8), additive (0.8–1.0) or antagonistic (>1.0). In addition, the cell cultures were treated with 100 µM 5-FU, used here as a positive control.

**Figure 4:**
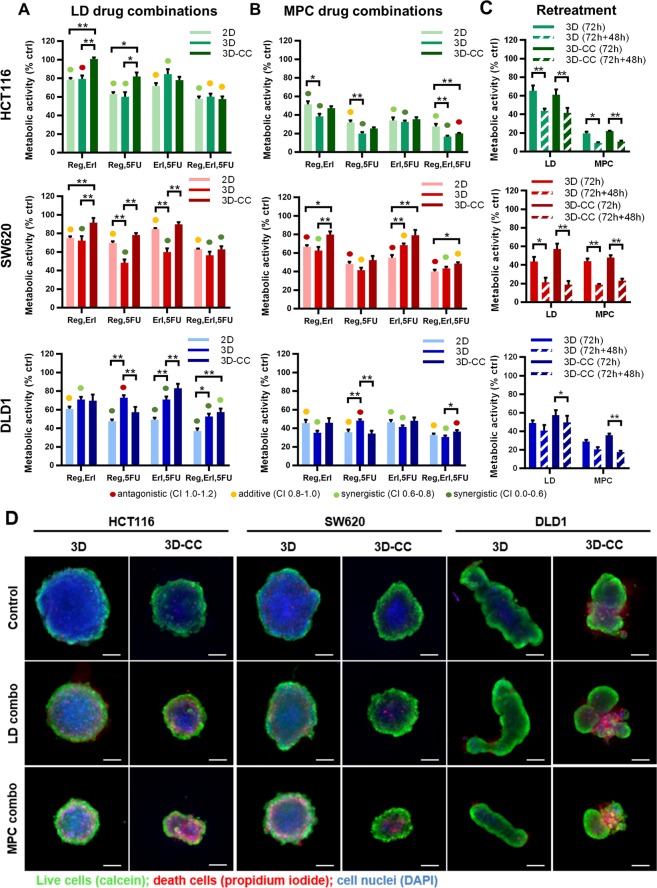
Drug combination activity in 2D and 3D (co-)cultures. Metabolic activity of HCT116, SW620 and DLD1 2D, 3D and 3D co-cultures (3D-CC) after treatment with drug combinations including regorafenib (reg), erlotinib (erl) and 5-fluorouracil (5-FU) administered at (**A**) optimized low doses (LD) or (**B**) maximum plasma concentrations (MPC). (**C**) Metabolic activity of HCT116, SW620 and DLD1 after double treatment on day 2 and day 5 post spheroid formation with LD or MPC drug combinations. Error bars represent the standard error of the mean (SEM) for n = 3–15. Significance of *p < 0.05, **p < 0.01 and ***p < 0.001 represent the comparison between 2D, 3D and 3D-CC as determined by a one-way ANOVA with post-hoc Dunnett’s multiple comparison test. (**D**) Representative images of CRC 3D–CCs of DLD1, SW620 and HCT116 taken at day 5, after 72 hours incubation with 3-drug combinations at low dose (LD) or at the maximal plasma concentration (MPC). Scale bar represents 200 µm.

3D co-cultures containing HCT116 cells were significantly less sensitive to low-dose 2-drug combinations containing regorafenib, as compared to 2D and 3D cultures, see Fig. 4A, upper panel, (**p < 0.01 for regorafenib + erlotinib and *p < 0.05 for regorafenib + 5-FU). The 3-drug combination was similarly active in all tested systems (approx. 40% activity), Fig. 4A. In contrast, at MPC doses the 2D cultures were less sensitive to both the 2-drug and 3-drug combinations containing regorafenib (*p < 0.05 for regorafenib + erlotinib and **p < 0.01 for both regorafenib + 5-FU and regorafenib + erlotinib + 5-FU). Drug combinations composed of both regorafenib and 5-FU at MPC had similar efficacy (approx. 80% of metabolic activity inhibition) compared to the CTRL (Fig. 4B, upper panel). The decrease in metabolic activity in the 3D homotypic cultures and 3D co-cultures correlated with a decrease in spheroid size and more pronounced cell death (Fig. 4D).

Variations in treatment efficacy were also present for SW620 cells. The SW620 3D co-cultures were significantly less sensitive to 2-drug combinations at both LD (**p < 0.01 for each of the 2-drug combinations) and MPC (*p < 0.05 for regorafenib + erlotinib and **p < 0.01 for erlotinib + 5-FU), see Fig. 4A,B, middle row. Although erlotinib presented synergistic interactions with other compounds, it had no activity as a single drug in SW620 cells at MPC (Fig. 2) and it had only a minor contribution to the overall efficacy of the drug combination. As such, the 3-drug combination was only slightly more active than the 2-drug combination composed of only regorafenib + 5-FU. Interestingly, the activity of the 3-drug combination was similar when at LD and MPC (Fig. 4A,B, middle row). The increase in drug concentration from LD to MPC for regorafenib and 5-FU also correlated with a shift from synergistic towards rather antagonistic drug interactions (Supplementary Table S4). In accordance with the drop in metabolic activity, an increase in cell death was observed in the LD- and MPC-treated spheroids and spheroid size was clearly smaller as compared to the control spheroids (Fig. 4D).

DLD1 spheroids displayed clear heterogeneous response profiles after treatment with LD drug combinations. 3D-CC and to a lesser extent the 3D cultures were significantly less sensitive to 2-drug or 3-drug combinations than 2D cultures (*p < 0.05 for 3D cultures treated with regorafenib + erlotinib and erlotinib + 5-FU; **p < 0.01 for 3D co-cultures treated with regorafenib + 5-FU, erlotinib + 5-FU and regorafenib + erlotinib + 5-FU), see Fig. 4A, third row. At MPC these differences were less apparent and the 2-drug combinations were almost as active as the 3-drug combinations, see Fig. 4B, third row. We observed unexpected activity of the drug combination containing erlotinib in DLD1 cells. Furthermore, inhibition of spheroid size versus untreated spheroids was observed (Fig. 4D). It is interesting to note that with the increase in dose the drug-drug interactions shifted from synergistic to additive (Supplementary Table S4). Taken together, differences in cell culture system affected drug sensitivity in a cell type and drug concentration specific manner. Furthermore, drug doses profoundly impacted the coordinated action, i.e. leading to unwanted antagonistic effects.

Since in clinical settings the CRC patients undergo repeated treatments, we investigated the effect of retreatment of the LD and MPC drug combinations on the cell viability of the 3D and 3D-CC systems. Here, the first 72 hours of drug incubation (day 2 to 5) was followed with subsequent retreatment (day 5 to 7) using the 3-drug combination, i.e. regorafenib + erlotinib + 5-FU. An increased treatment efficacy for each of the cell types treated with the LD or MPC drug combinations was observed (Fig. 4C). Interestingly, in both SW620 3D and 3D-CC two consecutive administrations of the LD drug combination led to approx. 80% activity. This was comparable to the activity of the MPC drug combination. Similar effect was obtained by a double treatment with 5-FU (positive control), which concentration was 10-fold or 100-fold higher than for MPC or LD drug combinations, respectively. Summarizing, we showed that the efficacy of low-dose drug combinations could be as high as of MPC drug combinations.

**Figure 5:**
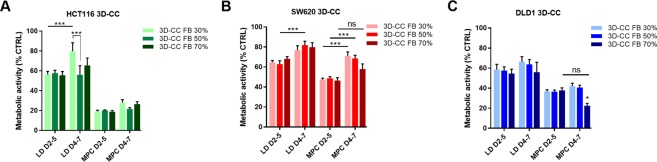
Efficacy of 3-drug combinations in 3D co-cultures with various fibroblast percentages. Metabolic activity of HCT116 (**A**), SW620 (**B**) and DLD1 (**C**) 3D co-cultures (3D-CC), consisting of tumor cells with 30%, 50% or 70% fibroblasts and an additinal 5% of endothelial cells, after treatment with 3-drug combinations of regorafenib, erlotinib and 5-fluorouracil at low dose (LD) or maximum plasma concentration (MPC). Treatment was performed for 72 h at day 2–5 or at day 4–7 after the start of spheroid formation. Error bars represent the standard error of the mean (SEM). Significance levels of *p < 0.05, **p < 0.01 and ***p < 0.001 were determined by a two-way ANOVA with post-hoc Sidak’s multiple comparison test.

CRC tumors are characterized with heterogeneous percentages of stroma with overall higher stroma in metastatic high grade CRC compared to low grade CRC^31^. To evaluate how the tumor:fibroblast ratio influences treatment efficacy of our drug combinations, we established 3D co-cultures with different compositions of tumor cells:fibroblasts consisting of 30%, 50% or 70% fibroblasts (FB 30%, FB 50%, FB 70%) with an additional 5% of endothelial cells remaining constant in corresponding optimized cell culture media (Supplementary Fig. S4A). The 3-drug combination administered at LD and MPC was selected for further comparison in two treatment schedules (treatment performed between day 2–5 or 4–7), see Fig. 5. The results were cell line specific, with exception of DLD1 3D-CC FB 70% (p < 0.05) and HCT116 3D-CC 30% FB (p < 0.001) treated with the LD drug combination, no significant difference in treatment efficacy between the two treatment schedules was observed for the 3D-CCs with the same tumor:fibroblast compositions (Fig. 5A). Comparing treatment efficacy between the two treatment schedules, we noted a significantly lower treatment efficacy of the drug combination applied at LD when treated for 72 h from day 4–7 for 3D-CC FB 30% (p < 0.001). This phenomenon was also observed in homotypic 3D cultures seeded with the same number of cells at both treatment schedules (Supplementary Fig. S5A, p < 0.05). Drug combinations applied at MPC induced a similar effect in HCT116 cells for both treatment schedules (Fig. 5A).

**Figure 6:**
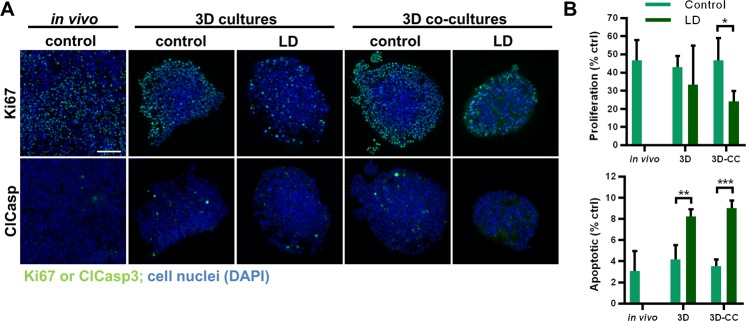
Morphology and viability of HCT116 3D (co)-cultures. (**A**) Representative images of cross-sections from the spheroid center and (**B**) quantification of proliferation (Ki67) and apoptosis (ClCasp3) of HCT116 3D and 3D co-cultures after treatment with control or LD drug combinations, as well as *in vivo* subcutaneously grown control tumors. Error bars represent the standard deviation. Significance of *p < 0.05, **p < 0.01 and ***p < 0.001 represent the comparison of the treated conditions with the untreated control as determined with an unpaired student’s t-test. Scale bar represents 100 µm for all images.

SW620 3D-CCs exposed to the LD drug combination were significantly less sensitive in the 3D-CC composition with 50% FB only between the two treatment schedules (Fig. 6B, p < 0.001). This treatment time-related effect was more prominent when treated with the MPC drug combination resulting in approx. 20% lower activity when treated from day 4–7, as compared to day 2–5 for 3D-CC with FB 30% and F 50% (Fig. 5B, p < 0.001). Similar behavior was observed in homotypic 3D cultures (Supplementary Fig. S5B, p < 0.01).

DLD1 3D-CCs in all tumor:fibroblast compositions tested had no differences in the treatment response between the two treatment schedules (Fig. 5C). In contrast, significant differences are observed in the homotypic 3D spheroids (Supplementary Fig. S5C). In general, the various CRC 3D co-cultures were more sensitive to drug combinations administered from day 4–7 than the homotypic 3D spheroids, emphasizing the necessity of testing the treatment efficacies in the heterotypic co-culture systems.

Spheroid metabolic activity was reproducible with an intraplate CV < 10% and interplate CV < 13% for day 5 spheroids (Supplementary Tables S5–S8). 3D co-culture spheroid size distribution is also narrow across experiments with an inter-experiment plate-to-plate variance of 7% < CV < 13% (depending on the cell line, Supplementary Tables S9, S10).

In order to confirm our results based on the measurements of metabolic activity and possibly observe the mechanism of cell growth inhibition, immunohistochemical staining for proliferation (Ki67, green) and apoptosis (cleaved caspase 3, ClCasp3, blue) was performed in selected conditions (CTRL and LD-drug combination treated HCT116 3D and 3D-CC spheroids). Ki67 positive (Ki67+) staining was present mostly at the spheroid periphery with only scarce intra-spheroid Ki67+ cells (Fig. 6A**)**. In the LD-treated spheroids, the Ki67^+^ staining was generally much lower than in the CTRL spheroids. Image-based quantification revealed that 3D and 3D-CC treated with LD drug combinations had lower expression of Ki67^+^ cells (*p < 0.05 for 3D co-cultures). Higher levels of apoptosis in LD-treated samples compared to CTRL were confirmed by cleaved-caspase-3 positive (ClCasp3^+^) staining in spheroid cross-sections (**p < 0.01 and ***p < 0.001, for 3D and 3D-CC, respectively), Fig. 6B. Non-specific localization was observed in the spheroids - apoptotic cells appeared randomly without clustering in specific areas. To conclude, the treatment with low-dose drug combinations decreased the cells proliferation and increased induction of apoptotic cell death.

### Signaling in 3D and 3D-CC upon AKT and MAPK pathway regulation and matrix deposition

Concerning the cellular signaling activity in the spheroids, we performed western blot analysis for detection of total and phosphorylated proteins in the MAPK and AKT-mTOR signaling pathways. The western blots and image-based quantifications are presented in Supplementary Figs S9 and S10. We observed that both signaling pathways are active in all three cell lines. In general, there was not an obvious difference between 3D and 3D-CC spheroids, in terms of cellular signalling pathways. Exposure to MPC inhibited the presence of total and phosphorylated MAPK, as well as AKT and pS6 proteins signaling molecules.

**Figure 7:**
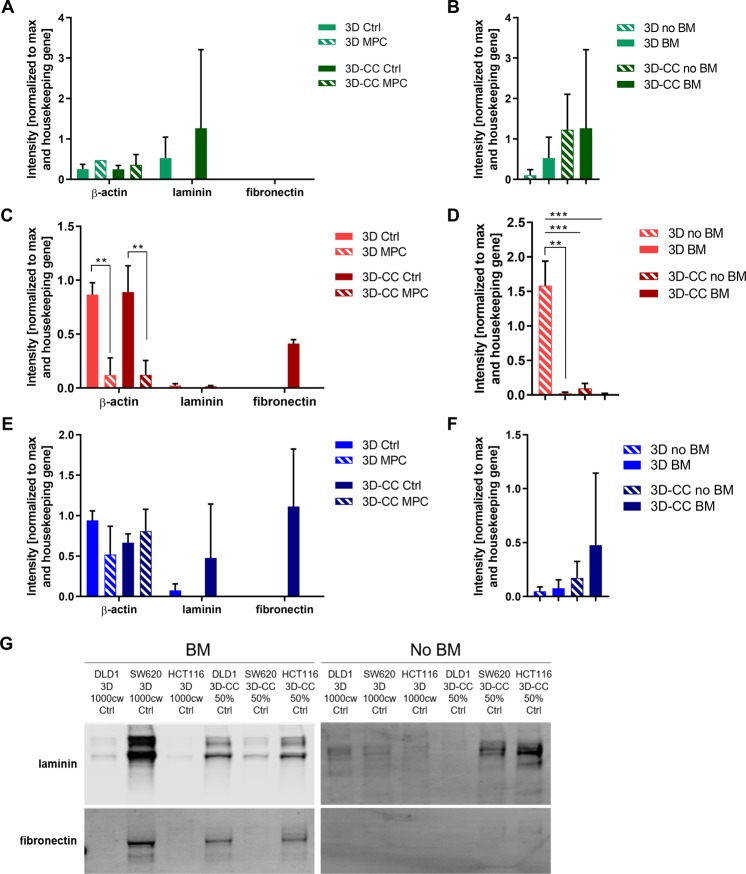
Detection of extracellular matrix components in the 3D and 3D co-cultures. (**A**,**C**,**E**) Extracellular matrix components laminin and fibronectin, as well as beta (β)-actin, were detected in control and treated 3D and 3D co-cultures of HCT116 (green), SW620 (red) DLD1 (blue) cells as indicated. (**B**,**D**,**F**) quantification of laminin protein expression of 3D and 3D-CC spheroids with and without 2.5% Matrigel^TM^(BM). Results are presented as mean values of N = 2–3 independent experiments with the error bars representing the standard deviation. Significances of **p < 0.01, ***p < 0.001 were determined with a two-way ANOVA test with post-hoc Tukey’s multiple comparison. (**G**) Representative western blots quantified in (**A**–**F**). All gels for western blot analysis were run under the same experimental conditions. 50% indicates 3D co-cultures of CRC cells with fibroblasts in ratio 1:1 and 5% endothelial cells. Unprocessed full blots can be found in Supplementary Fig. S10. The blots images were prepared in compliance with the digital image and integrity policies of the journal. Images were obtained with the Licor Odyssey CLx scanner at one default exposure setting.

In order to verify whether the co-culture microenvironment is involved in the deposition of extracellular matrix we performed western blot analysis of laminin and fibronectin production in 3D and 3D-CC cultures in the presence or absence of the drug combination at MPC. We selected the 3D-CCs containing 50% fibroblasts for further characterization as this is reported for high grade CRC and corresponds to the SW620 and DLD1 CRC cells. As our 3D and 3D co-cultures are supplemented with 2.5% Matrigel^MT^ basement membrane (BM) as discussed previously, we included control conditions without the addition of Matrigel^TM^ to determine if the CRC cells alone without those additional components can synthesize ECM proteins. Clearly, matrix is deposited in the spheroids. Co-cultures express more matrix proteins, presumably due to the presence of fibroblasts. In two of the three cell lines (not in HCT116) also fibronectin is deposited in co-culture spheroids (Fig. 7 and Supplementary Fig. S10).

## Discussion

In this study, we successfully established robust and reproducible short-term extra cellular matrix-based 3D cell culture systems for six colon cancer cell lines. To mimic the stromal compartment of tumors in the 3D cultures, human fibroblasts (FB) and endothelial cells (EC) were added. The efficacy of various single drugs and drug combinations was tested in the 3D co-cultures and compared with results in 2D and homotypic 3D cultures. Several interesting observations suggest that 3D co-cultures are more relevant, providing a higher level of translational information and enables more relevant patient-specific treatment options.

To establish our 3D spheroids, we selected a scaffold-free method to create controlled conditions allowing single, reproducible and assay-accessible 3D and 3D co-cultures to form. Various scaffold-free methods exist including hanging drops^32,33^ and magnetic levitation^34,35^. However, we selected to use low-attachment plates^27,28^ for ease in administration of drug combination treatments. Additionally, we incorporated ECM by seeding them in the presence of Matrigel^TM^ basement membrane reported to promote spheroid formation^28^. Matrigel^TM^ is derived from Engelbreth-Holm-Swarm (EHS) mouse sarcoma cells and is rich in EC components such as laminin, collagen, heparin sulfate proteoglycans and soluble factors, all present in the basement membrane of the colon and intestine. We supplemented the 3D cultures with only 2.5% Matrigel^TM^, sufficient to promote spheroid formation, but still allowing continued ease in handling and analyzing the various 3D cultures, and simultaneously decrease the effect of batch-to-batch variations.

Mimicking the CRC tumor microenvironment in 3D cultures is complex, as it involves multi-directional interactions between stromal cells and tumor cells. The stromal compartment in CRC can consist of 30–80%^11^ of the total tumor cell population. It is known that this percentage increases in patients having received adjuvant chemotherapy^36,37^. Another stromal compartment is the cells forming the tumor vasculature. Whereas the number of endothelial cells (EC) in healthy colon tissues can be 1–2% of the total cell population, this can be markedly higher, up to 10%, in tumor samples^38^. To develop a 3D culture system with *in vivo*/clinical relevance we used cell mixtures of CRC and FB in ratio 1:1 and 5% EC.

The morphology of our 3D homotypic CRC cultures is fully consistent with previously reported spheroids of corresponding cell lines^11^, but we found striking morphological changes in spheroid shape when the same cells were co-cultured with FBs and ECs. We observed heterogeneous, irregularly shaped spheroids for all 3D co-cultures, with multi-directionally connected outgrowths. It is considered that this difference can be an advantage for spheroid survival through an increase in surface area and improved exchange of oxygen and nutrients^39^. Also others have observed that the presence of fibroblasts correlates with elongated shapes of the spheroids, peripheral membrane ruffling and filopodia, presence of gap junctions and desmosomes, as well as elevated expression of collagen-I and TGF-β by the fibroblasts^40^. In another study, 3D breast cancer cell co-cultures with fibroblasts were reported to have highly heterogeneous shapes that were specific to the cell line, and self-organization of fibroblasts within the co-cultures reflected tissue-like characteristics^41,42^.

In addition to morphological changes, we also observed an induction in the production of ECM components fibronectin (SW620 and DLD1) in the 3D co-cultures. Fibronectin was 3D co-culture specific, indicating that fibroblasts contribute to the ECM environment and this can, at least in part, be dependent on the BM supplied in the cell culture conditions. On signaling level cancer associated fibroblasts (CAFs) in 3D co-cultures have also been linked to CRC cell migration and invasion, partly dependent on FGF-2 and FGFR signaling^43^. In tumors, CAFs and ECs are linked to changes in ECM rigidity through their secretion of collagens, fibrin and (proteo)glycans^44^. This increased rigidity is associated with promoting tumor growth, invasion and angiogenesis^45,46^. Importantly, the production of laminin and fibronectin observed in the 3D co-cultures was reduced after treatment with the drug combination at MPC, indicating an effect of the treatment on all cell types of the 3D co-cultures.

An interesting observation was that the included endothelial cells tended to localize close to the fibroblasts in the center of the spheroids formed by DLD1, SW620 and HCT116 cells. Similar observations were made previously in co-cultures with breast cancer cells^42^. Cross-talk between the tumor cells and fibroblasts induces the expression of angiogenic growth factors, such as VEGF and PDGF, leading to the promotion of endothelial cell motility and subsequent tumor angiogenesis, explaining these observations^47^. Interestingly, the spheroids of the 3D co-cultures were smaller by approx. 20% than the respective homotypic 3D cultures (Fig. 4D). It is known that the fibroblasts in *in vivo* conditions promote tumor cell proliferation^48^. However, cancer-associated fibroblasts have slower growth rates then cancer cells^41^, which might explain the obtained difference in size between the 3D and 3D co-cultures.

In our short-term cultures, we set the treatment initiation at day 2, when the spheroids were well defined and had a diameter of approx. 350–400 µM. Although considerations for variations in drug response between 2D and 3D cultures may include differences in drug penetration, drug gradients^49,50^, altered gene expression^11,51^, augmented survival signaling, DNA repair, pH and transporters associated with drug resistance^52–55^, it might also be correlate to the mechanism of action of the drug itself. The analysis of dose-response curves obtained in 2D and 3D homotypic cultures for regorafenib, erlotinib or 5-fluorouracil resulted in interesting observations (Supplementary Fig. S3). First, in SW620, and to a lower extent in DLD1 3D cultures the 5-FU treatment efficacy was significantly reduced, as compared to 2D cultures. In addition, 3D cultures have in general lower percentages of dividing cells, as compared to 2D cultures, which are mainly located in the peripheral layers of the spheroid. Indeed, drugs requiring active cell division for their activity, like 5-FU, were previously reported to have higher efficacy in 2D cultures, as compared to 3D cultures^56,57^.

Each of the selected drugs in our study has non-overlapping cellular mechanisms of action. Therefore, the activity of a drug combination depends largely on how well drugs complement each other - synergizing drugs can obtain high efficacy and therapeutic selectivity^58,59^. The 2-drug and 3-drug combinations tested in our study were mostly synergistic or additive when administered at low doses. We observed synergistic or additive drug-drug interaction for regorafenib + erlotinib combinations administered at low-dose, but this effect was maintained only at MPC in HCT116 cells. Of note, only the HCT116 cells are p53 wild-type and p53-upregulated modulator of apoptosis (PUMA) was previously reported as a chemosensitizer *in vitro* and *in vivo*^60^, putatively explaining the loss of synergy or additivity in DLD1 and SW620, but not HCT116 cells. This observation is in agreement with other recent studies where *RAS* wild-type and mutant CRC tumors were treated with a combination of erlotinib and bevacizumab^61^. Moreover, synergy between regorafenib and the anti-EGFR antibody, cetuximab, was previously reported *in vitro* and *in vivo*^62^, as well as in a phase I clinical trial^63^. Currently, regorafenib is approved for patients with refractory mCRC after chemotherapy as a single agent. The combination of regorafenib with 5-fluorouracil has shown some clinical benefit^64,65^ and showed efficacy in our 3D and 3D co-cultures. It is important to note that we observed variations in drug combination efficacy between the cell types, cell ratios and culture systems, but none of the drug combinations containing regorafenib, erlotinib and 5-fluorouracil tested in this study were able to fully inhibit cell viability at the tested drug doses. In this respect, we are currently working on the identification of multidrug combinations^29,66,67^ to identify synergistic drug mixtures with higher anti-tumor efficacy and selectivity.

## Conclusions

Taken together, our results demonstrate that the 3D and 3D CRC co-culture systems are robust, reproducible and well suited for drug (combination) efficacy studies. With the integration of fibroblasts and endothelial cells, we were able to mimic important features of the CRC microenvironment *in vivo*. The current report suggests that the implementation of 3D co-culture systems can significantly help in the process of drug discovery and efficacy testing. It is expected that these culture systems can significantly support clinical translation of drug regimens.

## Supplementary information

Supplementary Information

Supplementary movie S1

Supplementary movie S2

Supplementary movie S3

## Data Availability

The datasets used and/or analyzed during the current study are available from the corresponding author on reasonable request.
