# Supplementary material for: Short-term 3D culture systems of various complexity for treatment optimization of colorectal carcinoma
Source: Sci Rep. 2019 May 8;9:7103. doi: 10.1038/s41598-019-42836-0 (PMC6506470; doi:10.1038/s41598-019-42836-0)
Supplement: Supplementary file 1 — Supplementary Information [file 41598_2019_42836_MOESM1_ESM.pdf]

Supplementary Information

Short-term 3D culture systems of various complexity for treatment optimization of colorectal carcinoma

Marloes Zoetemelk[1,2], Magdalena Rausch[1,2], Didier J. Colin[3], Olivier Dormond[4], Patrycja Nowak-Sliwinska[1,2]*

[1]Molecular Pharmacology Group, School of Pharmaceutical Sciences, University of Geneva, University of Lausanne, 1211 Geneva 4, Switzerland; [2]Translational Research Center in Oncohaematology, 1211 Geneva 4, Switzerland; [3]Centre for BioMedical Imaging (CIBM), University Hospitals and University of Geneva, 1211 Geneva 4, Switzerland; [4]Department of Visceral Surgery, Lausanne University Hospital, Lausanne, Switzerland

Supplementary Methods
Supplementary Figures S1-S9
Supplementary Tables S1-S10
Supplementary Movies S1-S3

Supplementary Methods

Immunohistochemistry

To unmask cellular targeted markers glass slides were heated in a beaker with TRIS/EDTA until boiling and continued to be heated for an additional 15 min on the defrost program after which they were left to cool. Before blocking, slides were washed with PBS and PBS supplemented with 0.2% Triton-X (PBST-X). To perform blocking, slides were covered with 80 µL PBST-X and 1% BSA blocking buffer and incubated for 20 min at RT. Following, cells were stained overnight at 4 °C with primary antibodies, i.e. Ki67 Rabbit mAb (9027, Cell Signaling) or CleavedCaspase3 Rabbit mAb (9664, Cell Signaling). Slides were washed twice with PBST-X and once with PBS and incubated for 1 hour at RT in the dark with the secondary antibody AF488 Donkey-α-Rabbit (A-21206, ThermoFisher Scientific) and DAPI. Slides were washed twice with PBST-X, washed once with PBS and mounted with Dako fluorescence mounting medium (S3023, Agilent) for fluorescence imaging.

Supplementary Figure S1

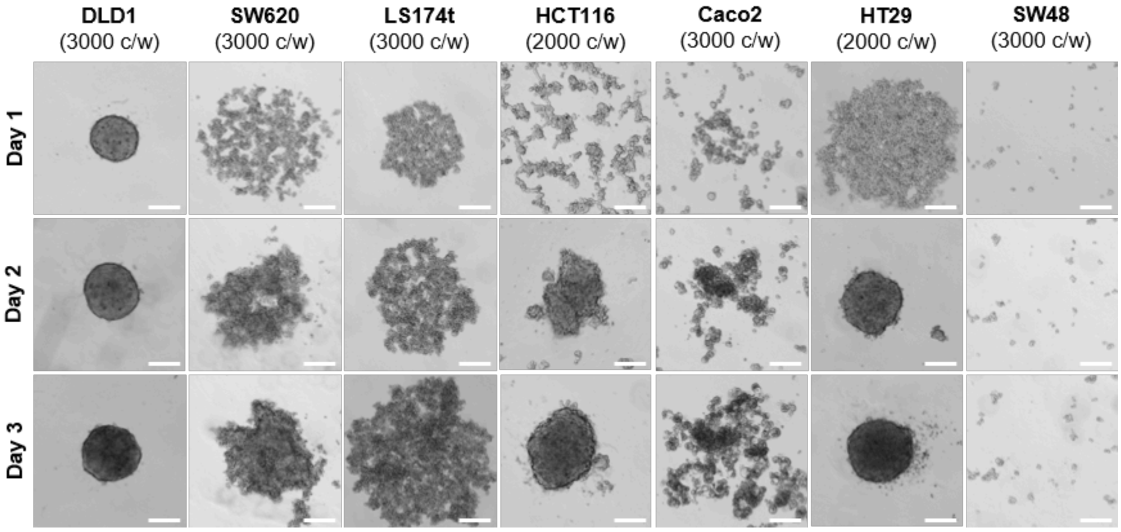

Supplementary Figure S1. CRC spheroid kinetics and morphology.
Representative images of CRC cells seeded in low-attachment plates without 2.5% basement membrane. Seeding densities of spheroid forming cell lines DLD1, SW620 and HT29 optimized to approximately 400 µm in diameter at day 2. Scale bar represents 200 µm.

Supplementary Figure S2

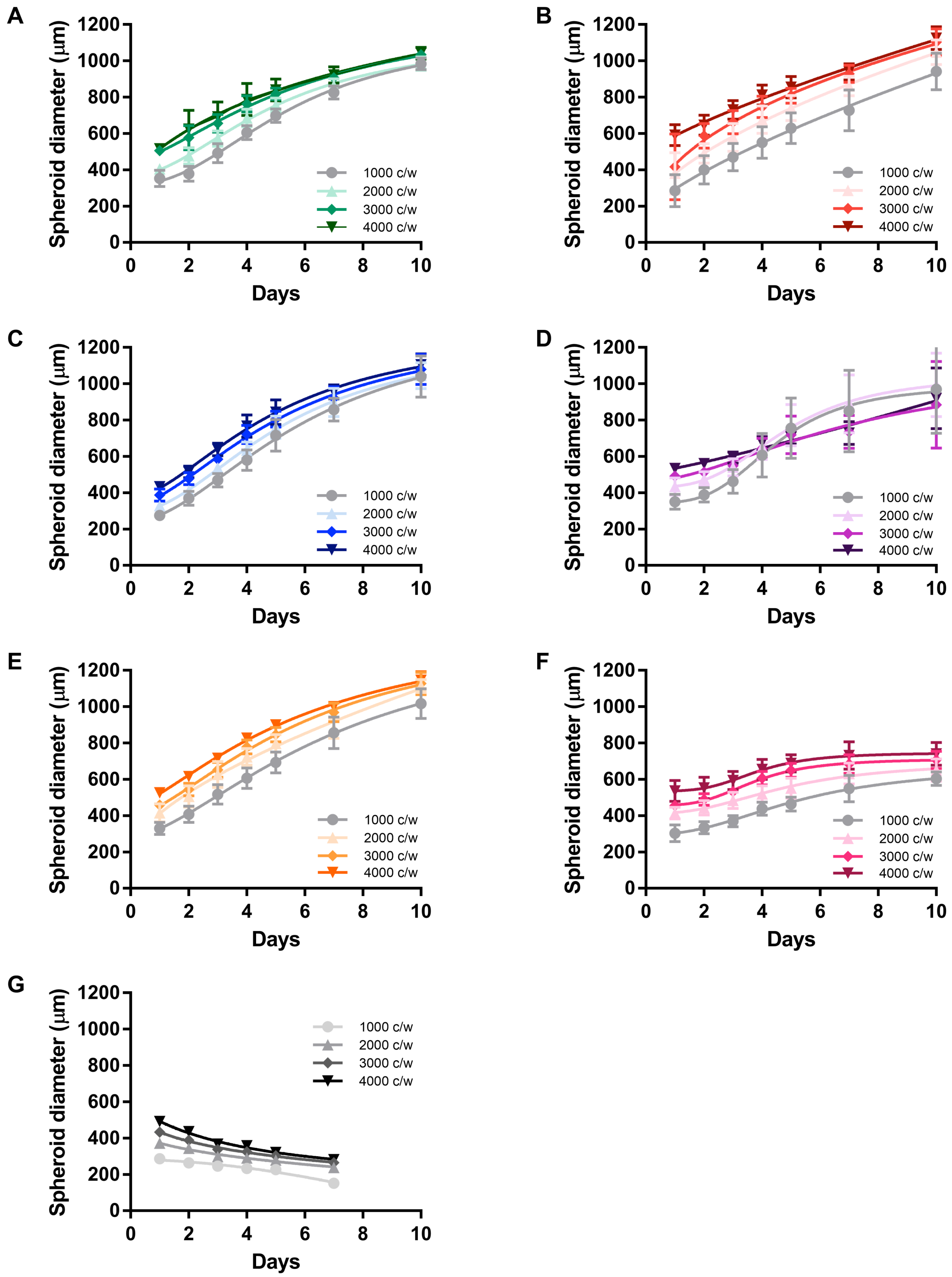

Supplementary Figure S2. Spheroid seeding density and size change in time.
Growth kinetics of spheroid diameter of **A** HCT116, **B** DLD1, **C** SW620, **D** HT29, **E** LS174T, **F** Caco2 and **G** CCD841 from day 1 to 10 seeded 1000-4000 c/w. Error bar represents the standard deviation of n = 3-15.

Supplementary Figure S3

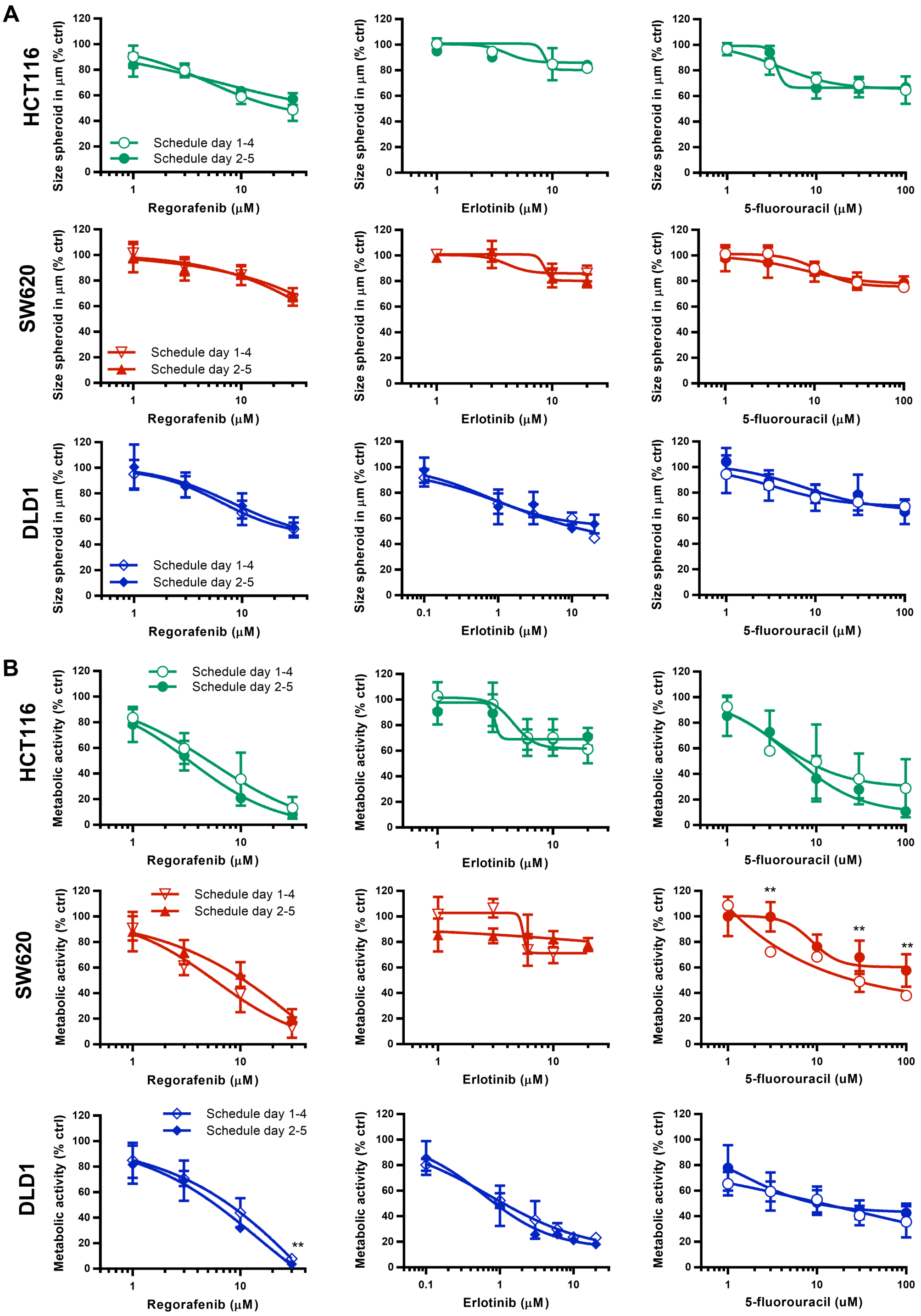

Supplemantary Figure S3. Dose-dependent regorafenib, erlotinib or 5-fluorouracil sensitivity in CRC 3D cultures.

A Spheroid size and **B** metabolic activity as a function of drug doses in HCT116, SW620 and DLD1 3D cultures were treated for 72 hours with dose ranges of regorafenib (1-30 µM), erlotinib (0.1-20 µM) or 5-fluorouracil (1-100 µM). Dose-response curves were obtained for each drug administered for 72 hours at two treatment schedules, i.e. (i) 1 day after spheroids were formed (Figure 2B, open symbols), or (ii) 2 days after spheroid formation (closed symbols). Error bars represent the standard deviation (spheroid size measurements n = 3-9; metabolic activity measurements n = 6-21). Significances of *p < 0.05 and **p < 0.01 define the differences for each drug concentration between the treatment schedules as determined with the unpaired multiple T-test.

Supplementary Figure S4

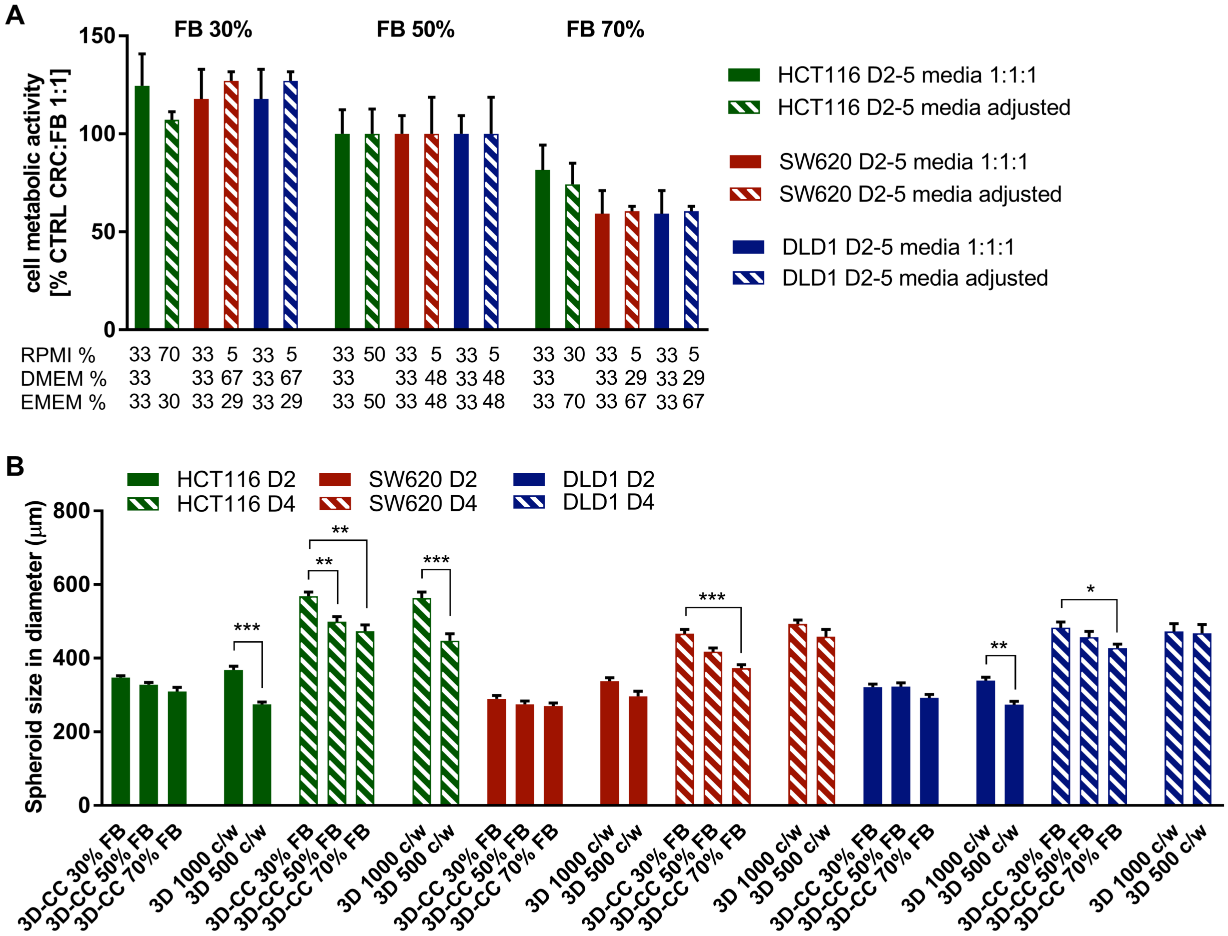

Supplemantary Figure S4. Optimization of 3D spheroid growth conditions.
A Metabolic activity of HCT116, SW620 and DLD1 3D co-cultures consisting of tumor cells with 30%, 50% or 70% fibroblasts and an additinal 5% of endothelial cells relative to the 3D co-cultures with 50% fibroblasts at 100%, tested in various media compositions (DMEM, RPMI and/or EMEM), as indicated. Error bars represent the standard deviation of n = 3. **B** Spheroid size of HCT116, SW620 and DLD1 3D cultures at 1000 or 500 c/w and 3D co-cultures with various percentages of fibroblasts at the start of treatment at day 2 and day 5. Error bars represent the standard error of the mean of n = 9. Significances of *p < 0.05, **p < 0.01 and ***p < 0.001 define the differences between the CRC 3D and 3D co-culture conditions as determined with a two-way ANOVA with post-hoc Tukey`s multiple comparisons test.

Supplementary Figure S5

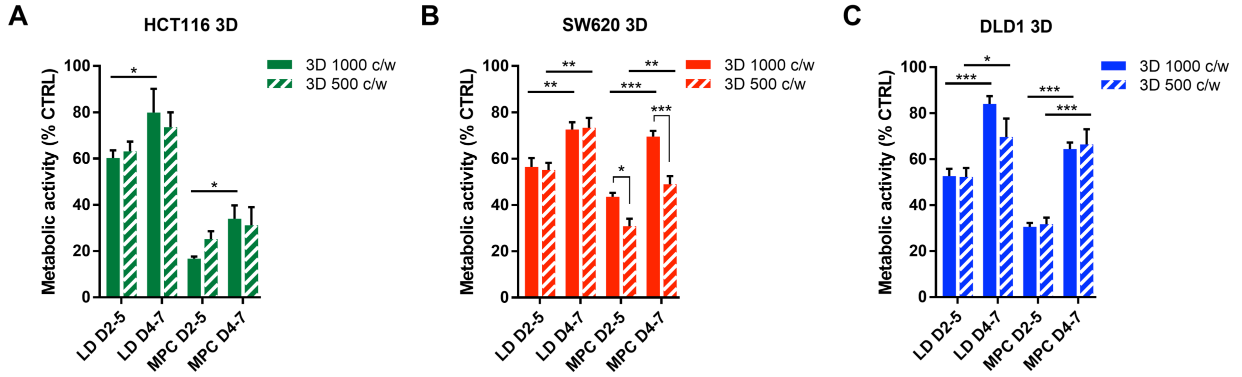

Supplemantary Figure S5. Responsiveness of 3D co-culture spheroids to different treatment schedules.

Metabolic activity of HCT116 (**A**), SW620 (**B**) and DLD1 (**C**) 3D cultures at 1000 or 500 c/w after treatment with the low dose (LD) and max plasma concentration (MPC) drug combinations in two treatment schedules from day 2-5 and day 4-7. Error bars represent the standard deviation of n = 9-12. Significances of *p < 0.05, **p < 0.01 and ***p < 0.001 represent the comparison between the 3D cultures and treatment schedules as determined with a two-way ANOVA with post-hoc Sidak`s multiple comparisons test.

Supplementary Figure S6

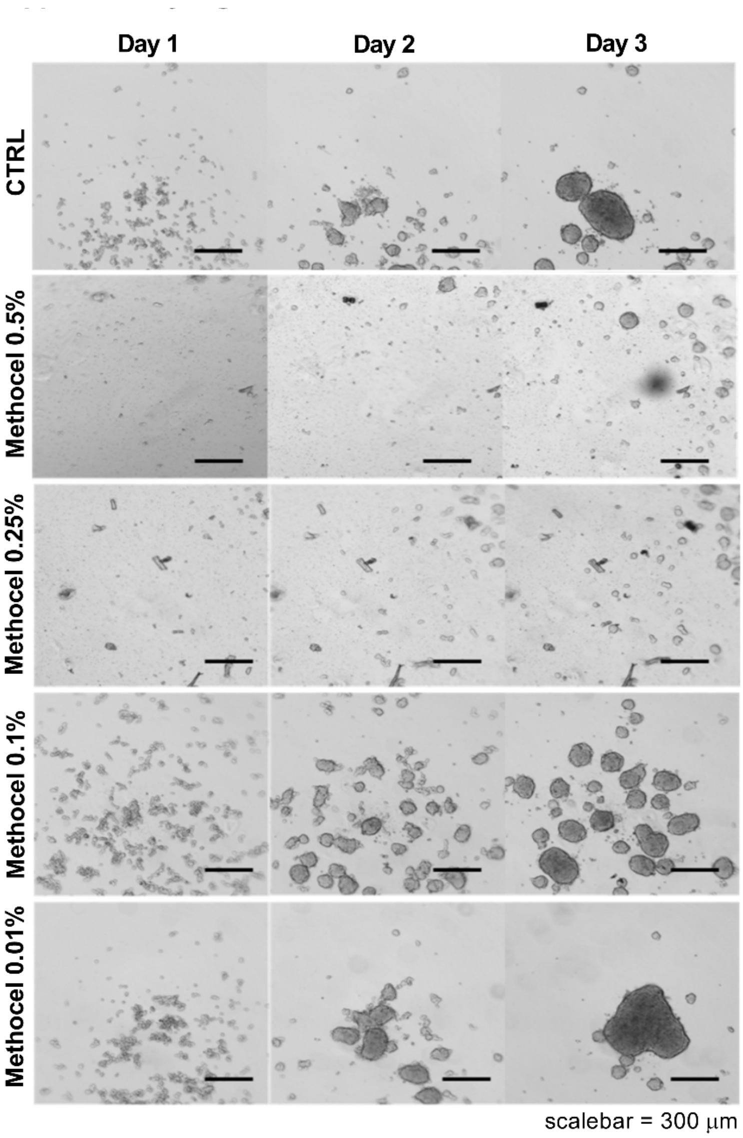

Supplementary Figure S6. HCT116 spheroid formation with methylcellulose supplement. Representative images of CRC cells seeded in low-attachment plates supplemented with Methocel methylcellulose at 0.01%, 0.1%, 0.25% and 0.5%. Scale bar represents 300 µm.

Supplementary Figure S7

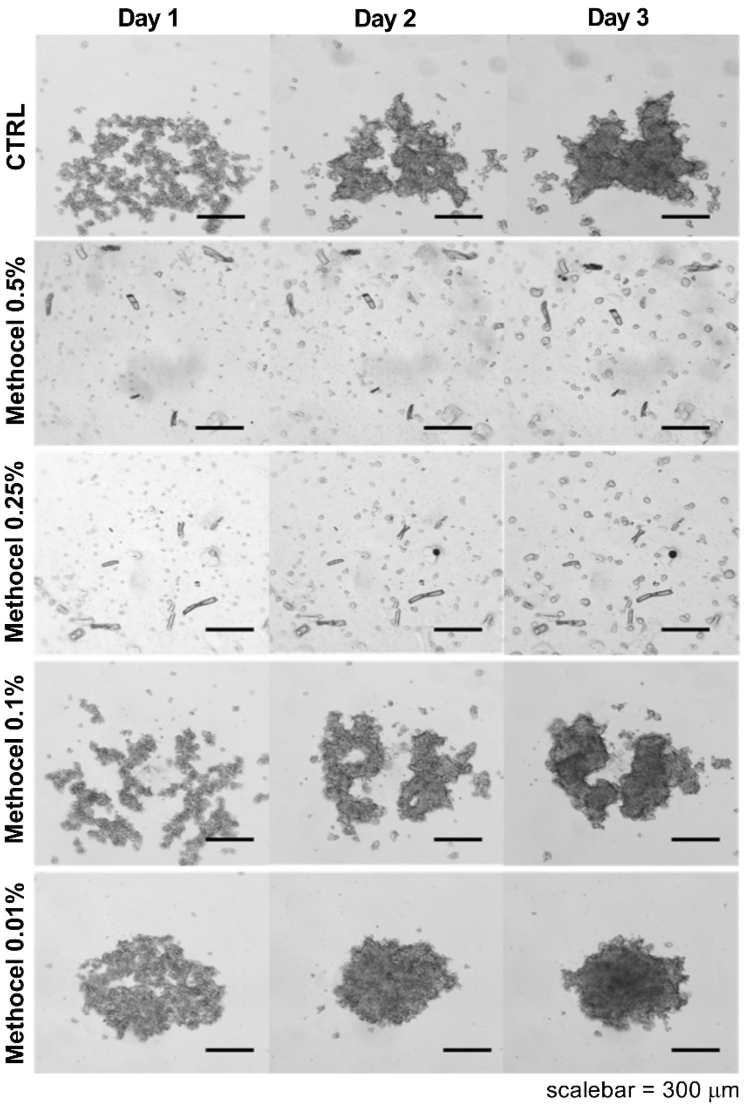

Supplementary Figure S7. SW620 spheroid formation with methylcellulose supplement. Representative images of CRC cells seeded in low-attachment plates supplemented with Methocel methylcellulose at 0.01%, 0.1%, 0.25% and 0.5%. Scale bar represents 300 µm.

Supplementary Figure S8

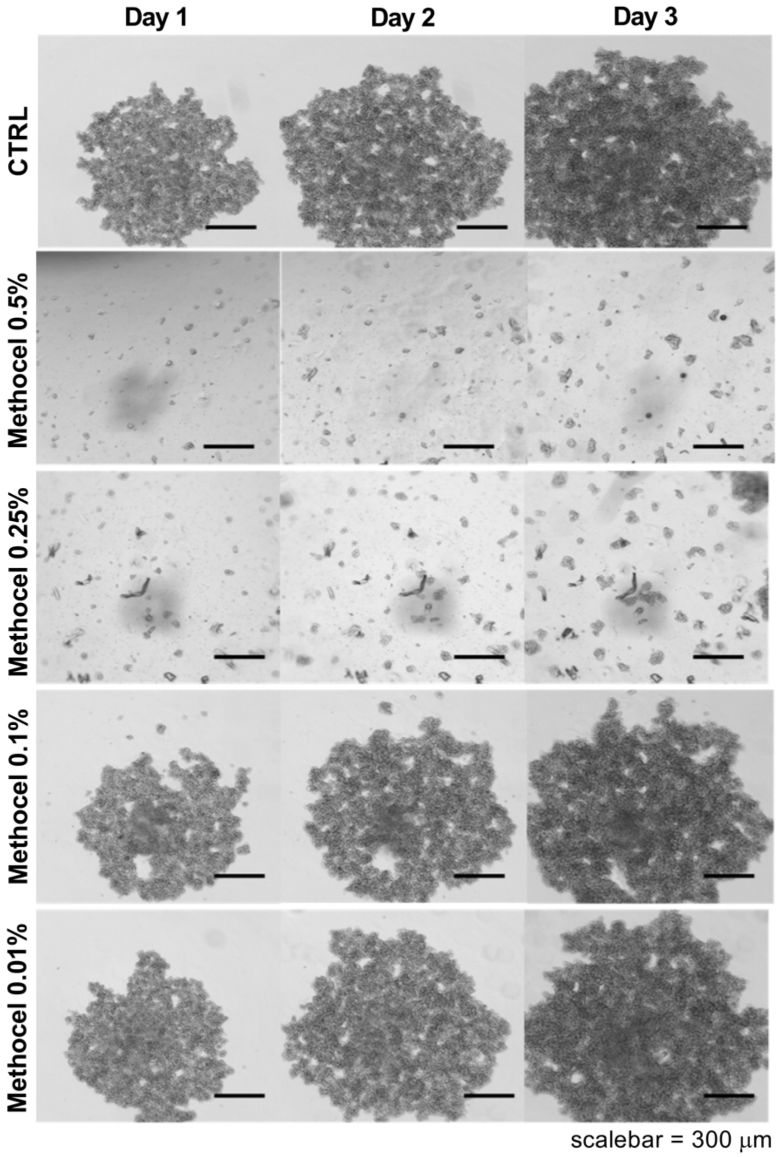

Supplementary Figure S8. LS174T spheroid formation with methylcellulose supplement. Representative images of CRC cells seeded in low-attachment plates supplemented with Methocel methylcellulose at 0.01%, 0.1%, 0.25% and 0.5%. Scale bar represents 300 µm.

Supplementary Figure S9

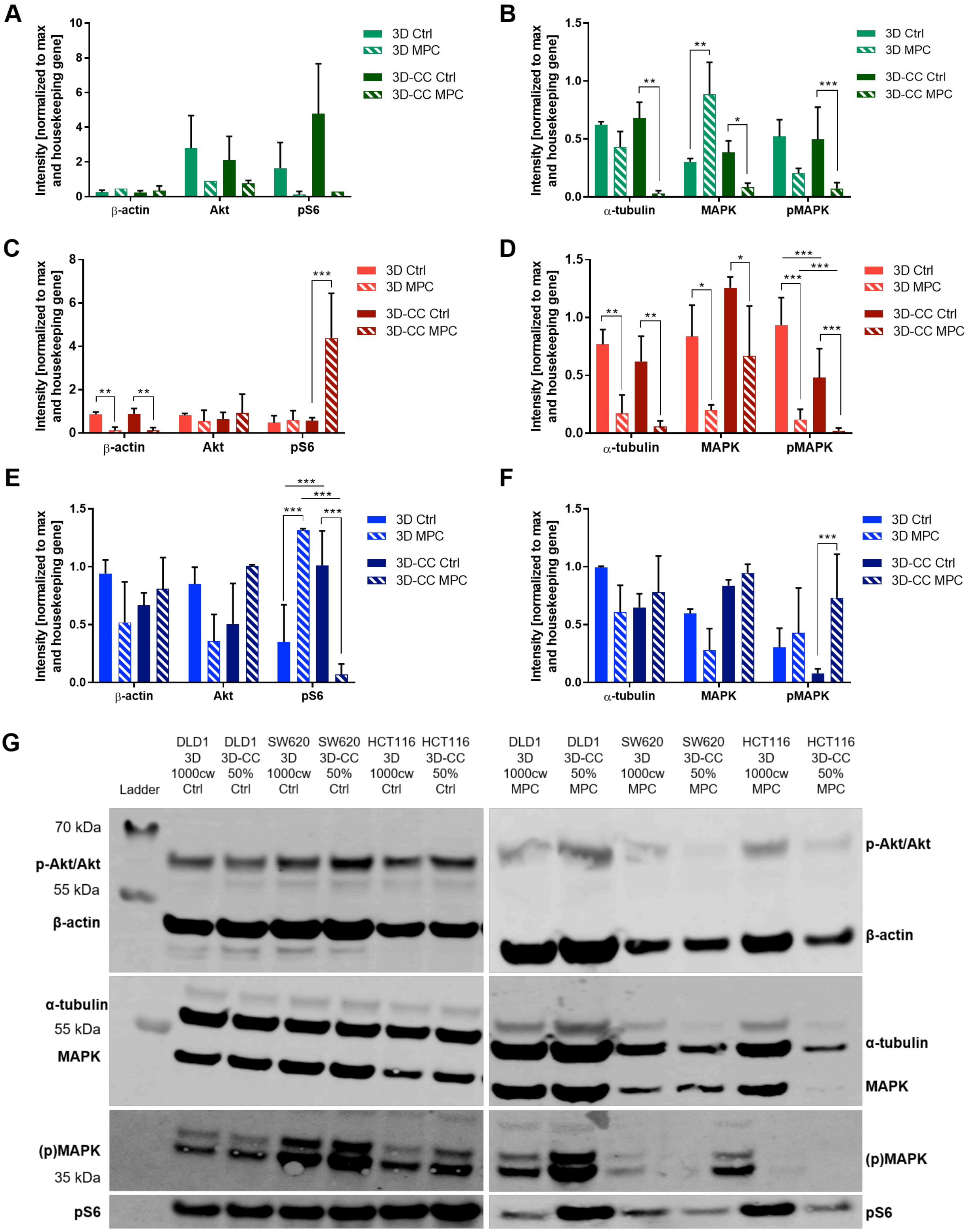

Supplementary Figure S9. PI3K-AKT-mTOR and RAS-RAF-MAPK signaling in 3D and 3D co-cultures for three CRC cell lines.
Western blot analysis of various proteins and phosphorylated (p-) proteins in the PI3K-AKT-mTOR and RAS-RAF-MAPK pathway of HCT116, SW620 and DLD1 3D cultures (seeded 1000 c/w) and 3D co-cultures (3D-CC, seeded with 50% fibroblasts). Cultures were treated with a 3-drug combination at maximum plasma concentration (MPC) or with CTRL. Analysis of **A,C,E** β-actin, Akt and p-rpS6 (S235/236), and **B,D,F** α-tubulin, MAPK and p-MAPK (T202/Y204) in HCT116 (green), SW620 (red) and DLD1 (blue), respectively. Alfa (α)-tubulin and beta (β)-actin were used as loading

controls. The error bars represent the standard deviation over the mean. Significances of *p<0.05, **p<0.01, ***p<0.001 were determined with a two- way Anova test with post-hoc Tukey's multiple comparison.

Supplementary Figure S10

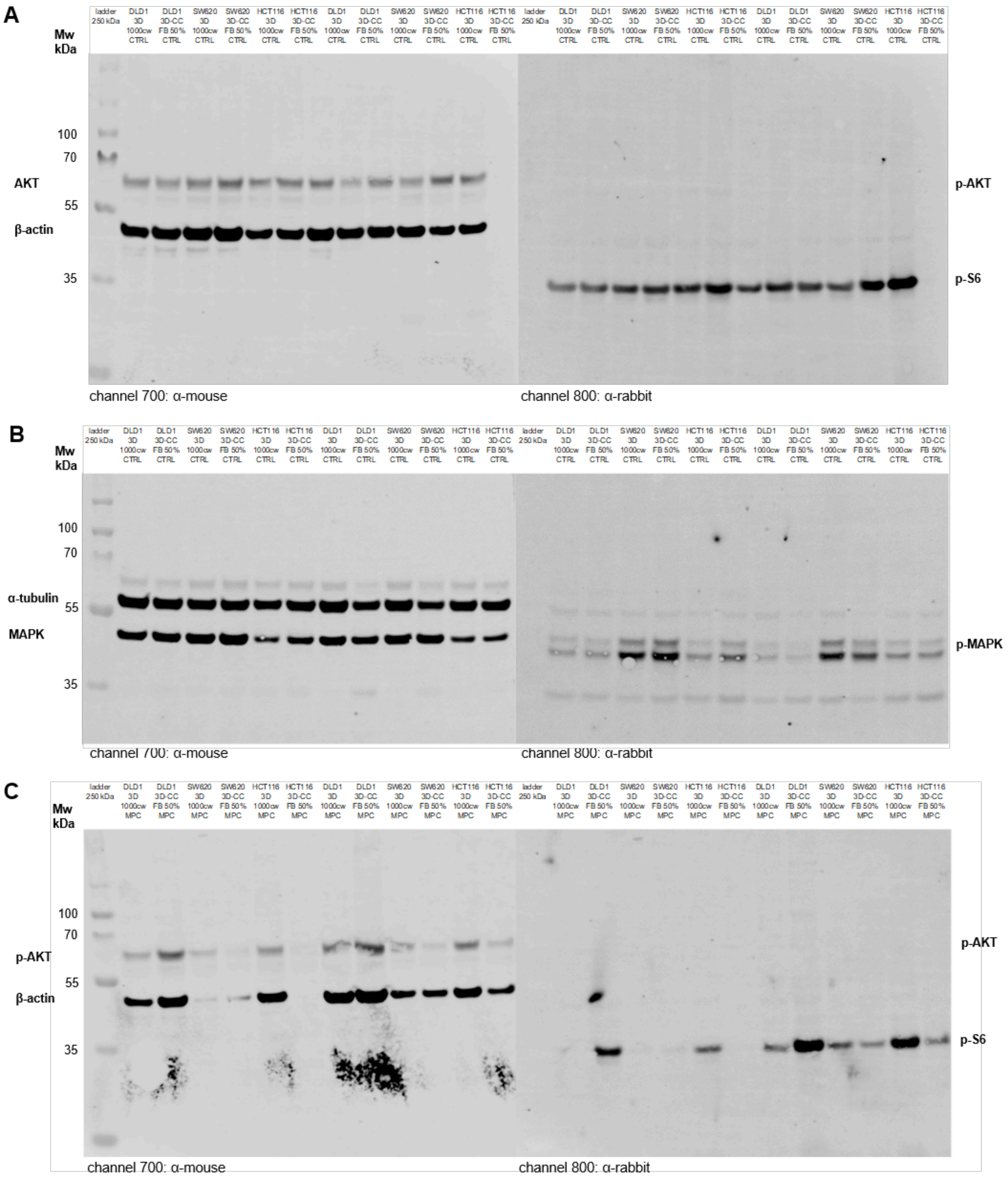

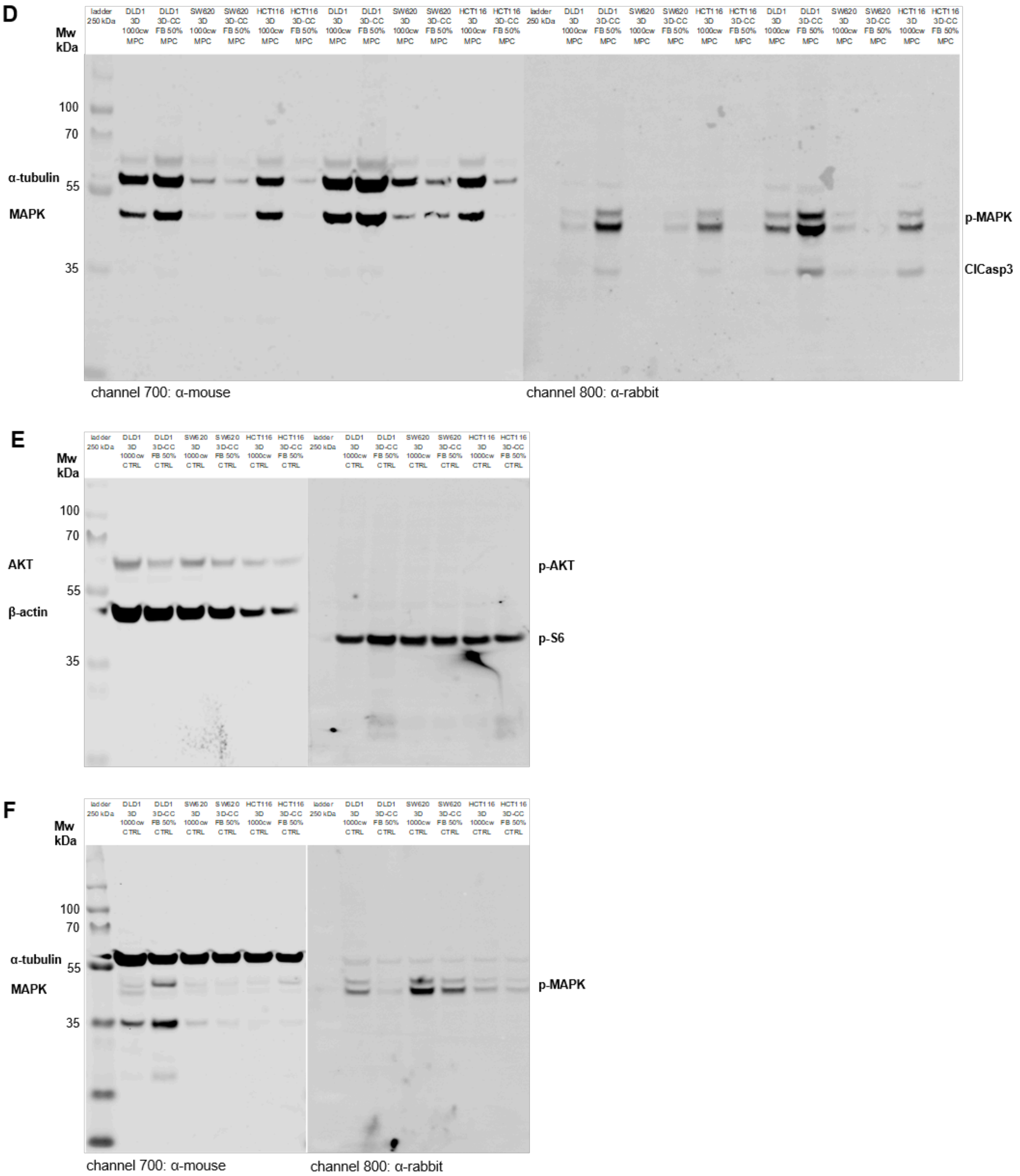
D
Mw kDa
ladder 250 kDa
DLD1 3D 1000cw MPC
DLD1 3D-CC FB 50% MPC
SW620 3D 1000cw MPC
SW620 3D-CC FB 50% MPC
HCT116 3D 1000cw MPC
HCT116 3D-CC FB 50% MPC
100
70
55
35
α-tubulin
MAPK
p-MAPK
ClCasp3
channel 700: α-mouse
channel 800: α-rabbit
E
Mw kDa
DLD1 3D 1000cw CTRL
DLD1 3D-CC FB 50% CTRL
SW620 3D 1000cw CTRL
SW620 3D-CC FB 50% CTRL
HCT116 3D 1000cw CTRL
HCT116 3D-CC FB 50% CTRL
AKT
β-actin
p-AKT
p-S6
F
Mw kDa
α-tubulin
MAPK
p-MAPK
channel 700: α-mouse
channel 800: α-rabbit

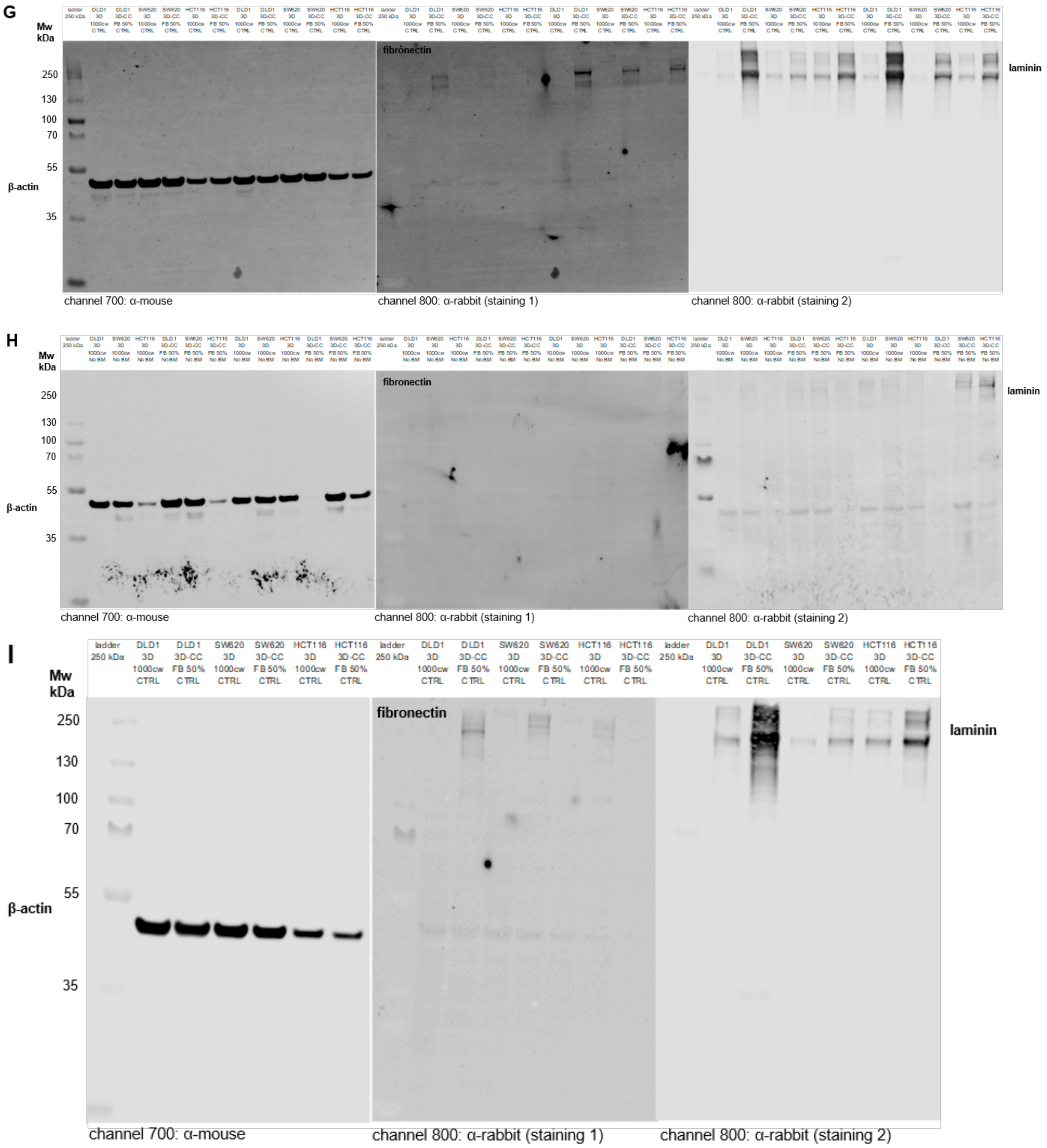

Supplementary Figure S10. Full length unprocessed western blot results of PI3K-AKT-mTOR and RAS-RAF-MAPK signaling and extracellular matrix in 3D and 3D co-cultures for three CRC cell lines.

Western blot analysis of various proteins and phosphorylated (p-) proteins in the PI3K-AKT-mTOR and RAS-RAF-MAPK pathway of HCT116, SW620 and DLD1 3D cultures (seeded 1000 c/w) and 3D co-cultures (3D-CC, seeded with 50% fibroblasts). Cultures were treated with a 3-drug combination at maximum plasma concentration (MPC) or with CTRL. **A,C,E** Western blots for β-actin (45 kDa), Akt (60 kDa) and p-rpS6 (S235/236, 32 kDa). **B,D,F** Western blots for α-tubulin (52 kDa), MAPK and p-MAPK (T202/Y204, 42 and 44 kDa) proteins. **G,H,I** Western blots for β-actin (45 kDa), fibronectin (285 kDa) and laminin (210 and 400 kDa) proteins. Cropped parts of the gels are presented in Figure 7. The blots images were prepared in compliance with the digital image and integrity policies of the journal. Images were obtained with the Licor Odyssey CLx scanner at one default exposure setting.

Supplementary Tables

Supplementary Table S1. Morphological characteristics of the 3D cultures

Cell line	*Morphology observed as of day two*
DLD1	Circular, compact core and defined periphery<br>Over time evolves into heterogeneous and lobular shapes
SW620	Spherical, clear and uncompact, no defined periphery<br>Over time increased compactness of the core
HCT116	Circular, compact core and fully defined periphery<br>Over time increased compactness of the core with loose cells surrounding the clearly defined rim
LS174T	Spherical form with defined periphery. Increased compactness of the core over time and increased definition of the periphery in slightly lobular shapes
HT29	Circular, compact core and defined periphery. Spheroid disintegration starting at day five
Caco2	Round, loose with varying shapes and defined periphery.<br>Over time increased compactness of the core
CCD841 CoN	Circular, clear and uncompact with aligned periphery at day one. From day 2 decreased size and compactness with cells sprouting at the periphery

* Circularity: the closer to a circularity of 1, the more symmetrical the spheroid.

Supplementary Table S2. Primary and secondary antibodies

**Primary Ab**	**Source**	**Cat#**	**Dilution**	**Host**
Akt pan	Cell Signaling	9272	750x	Rabbit
p- Akt (S473)	Cell Signaling	4060	300x	Rabbit
ERK1/2	Cell Signaling	4696	1000x	Mouse
p- ERK1/2 (T202/Y204)	Cell Signaling	9101	750x	Rabbit
p- rpS6 (S235/236)	Spring Bioscience	M3052	2000x	Rabbit
laminin	Abcam	ab11575	1000x	Rabbit
fibronectin	Abcam	AB2413	1000x	Rabbit
alfa-tubulin	Cell Signaling	3873S	1000x	Mouse
actin	Sigma	A2228	2000x	Mouse
**Secondary Ab**	**Source**	**Cat#**	**Dilution**	**Host**
Alexa Fluor® **680** goat anti-**Mouse**	Thermofisher	A21058	4000x	Mouse
IRDye **800** sheep anti-**Rabbit**	Rockland	611-632-122	4000x	Rabbit

Supplementary Table S3. $EC_{50,}$ low dose (LD) and maximal plasma concentration (MPC) for regorafenib, erlotinib and 5-flourouracil administered in HCT116, SW620 and DLD1 cells for 72 hours

		regorafenib (μM)			*erlotinib (μM)*			*5-fluorouracil (μM)*		
		EC_{50}	*LD*	*MPC(78)*	*EC_{50}*	*LD*	*MPC*	*EC_{50}*	*LD*	*MPC(79)*
**HCT116**	2D	>100	0.8	4.0	12.3	1.6	1.6	4.5	3.0	10.0
	3D	3.3	1.0	4.0	5.5	1.6	1.6	6.9	1.0	10.0
**SW620**	2D	5.7	3.0	4.0	10.1	1.6	1.6	8.0	3.0	10.0
	3D	6.8	1.0	4.0	nc	1.6	1.6	9.2	3.0	10.0
**DLD1**	2D	7.6	2.0	4.0	14.9	1.6	1.6	5.3	3.0	10.0
	3D	14.8	2.0	4.0	0.6	0.1	1.6	1.4	1.0	10.0

Note that if the EC_{20} was not below the MPC, the MPC was used in the LD combinations as well. EC_{50}: effective concentration inhibiting 50% of the cells, determined using a four-parameter non-linear fit for each drug; MPC: maximal plasma concentration of the drug in patients after clinical treatment; LD: low dose of each drug with approximately 20% activity; conc: concentration; nc: the non-linear dose response curve was not converged.

Supplementary Table S4. Drug combination activity in HCT116, SW620 or DLD1 in 2D and 3D cell (co-)cultures

Treatment		*LD*					*MPC*					*100 µM*	
		1x				*2x**	*1x*				*2x**	*1x*	*2x**
regorafenib		+	+		+	+	+	+		+	+		
erlotinib		+		+	+	+	+		+	+	+		
5-flourouracil			+	+	+	+		+	+	+	+	+	+
**HCT116**	**2D**	78.6	63.3	71.9	58.0		51.5	32.0	34.0	27.6		28.3	
	SEM	1.9	1.9	2.9	2.6		3.3	2.2	3.4	2.8		6.1	
	CI	0.66	0.75	0.87	0.67		0.54	0.83	0.74	0.72			
	**3D**	79.0	60.3	84.6	65.3	43.9	38.5	20.0	32.7	19.3	9.2	11.7	9.6
	SEM	3.8	4.9	5.2	5.7	2.4	2.9	1.6	1.4	1.1	0.7	1.0	0.5
	CI	1.13	0.61	0.61	0.9		0.5	0.26	0.38	0.25			
	**3D-CC**				61.2	41.6				9.2	10.5	10.0	7.4
	SEM				5.7	5.4				1.7	1.3	0.7	0.8
	CI				0.80					1.50			
**SW620**	**2D**	75.1	69.8	84.7	63.3		66.8	48.2	54.9	40.0		31.7	
	SEM	1.8	1.8	1.1	0.5		2.3	2.3	3.1	2.1		0.6	
	CI	0.82	0.91	0.76	0.80		1.22	1.22	1.32	1.15			
	**3D**	72.3	48.5	60.0	43.6	21.5	62.6	41.5	68.5	44.2	18.8	47.3	35.2
	SEM	4.8	3.4	4.0	5.2	5.0	3.9	2.8	1.7	2.6	0.7	2.4	2.7
	CI	0.37	0.22	0.20	0.20		0.61	0.94	0.98	0.72			
	**3D-CC**				57.1	18.7				47.9	23.2	34.1	19.4
	SEM				5.9	4.2				2.7		2.7	1.2
	CI				0.43					0.98	2.1		
**DLD1**	**2D**	60.8	47.6	49.3	37.0		45.6	35.8	46.5	32.8		27.5	
	SEM	2.4	2.1	2.9	2.9		3.6	3.0	2.5	1.6		4.1	
	CI	0.81	0.33	0.33	0.47		0.87	0.91	0.73	0.86			
	**3D**	71.1	73.1	71.0	48.8	40.7	35.2	48.2	41.5	28.8	20.3	43.7	28.0
	SEM	2.7	2.6	3.1	3.0		2.2	1.4	1.5	1.9	2.8	2.1	3.0
	CI	0.78	1.08	0.47	0.49	5.9	0.68	1.49	0.67	0.75			
	**3D-CC**				57.3	49.5				35.8	17.7	26.1	13.4
	SEM				5.6	7.1				1.7	1.2	6.8	0.6
	CI				0.72					2.00			

* Treatment of 3D (co-)cultures performed on day 2 and day 5 post spheroid formation. LD: low dose concentrations; MPC, maximal plasma concentrations clinically attained after treatment – see Figure 5 for exact drug doses; SEM: standard error of the mean; CI: combination index.

Supplementary Table S5. Calculation of intra- and interplate deviations of cell metabolic activity of HCT116 3D co-cultures with a composition of tumor:fibroblast of 50%:50%.

	HCT116 3D co-cultures metabolic activity (raw data)				
	tumor:fibroblast 50:50				
**Exp1**	**Ctrl**	**MPC**	**M1**	**M2**	**M3**
Result 1	71562	32919	19657	28184	14818
Result 2	78116	29241	16721	25379	13157
Result 3	88228	33650	16313	28632	15097
**Mean**	79302.0	31936.7	17563.7	27398.3	14357.3
**STD**	6855.35	1929.34	1489.55	1439.55	856.37
**%CV**	8.64	6.04	8.48	5.25	5.96
**Exp2**	**Ctrl**	**MPC**	**M1**	**M2**	**M3**
Result 4	68294	35143	18834	29200	15528
Result 5	62732	38706	20478	24647	19821
Result 6	79906	37543	17969	27509	15088
**Mean**	70310.7	37130.7	19093.7	27118.7	16812.3
**STD**	7154.80	1483.52	1040.62	1879.13	2135.02
**%CV**	10.18	4.00	5.45	6.93	12.70
**Exp3**	**Ctrl**	**MPC**	**M1**	**M2**	**M3**
Resul 7	92259	25633	10833	29249	15633
Resul 8	87591	27179	16154	29139	12913
Resul 9	86069	24379	11280	29888	15331
**Mean**	88639.7	25730.3	12755.7	29425.3	14625.7
**STD**	2633.60	1145.17	2409.90	330.22	1217.30
**%CV**	2.97	4.45	18.89	1.12	8.32
**Exp4**	**Ctrl**	**MPC**	**M1**	**M2**	**M3**
Result 10	70747	21639	12157	23378	10574
Result 11	90944	23066	10226	20172	21244
Result 12	75513	35868	18828	18379	18609
**Mean**	79068.0	26857.7	13737.0	20643.0	16809.0
**STD**	8620.06	6397.85	3685.19	2067.83	4538.15
**%CV**	10.90	23.82	26.83	10.02	27.00
**Exp5**	**Ctrl**	**MPC**	**M1**	**M2**	**M3**
Result 13	71562	14818	19657	28447	27182
Result 14	78116	13157	16721	26996	27771
Result 15	88228	15097	16313	29984	27168
**Mean**	79302.0	14357.3	17563.7	28475.7	27373.7
**STD**	6855.35	856.37	1489.55	1220.01	281.02
**%CV**	8.64	5.96	8.48	4.28	1.03
Exp6	**Ctrl**	**MPC**	**M1**	**M2**	**M3**
Result 16	43333	10743	13134	42448	21271
Result 17	45393	13475	12739	38427	22543
Result 18	31172	11954	13370	30305	24957
**Mean**	39966.0	12057.3	13081.0	37060.0	22923.7
**STD**	6274.91	1117.73	260.32	5050.72	1528.69
**%CV**	15.70	9.27	1.99	13.63	6.67
**Exp7**	**Ctrl**	**MPC**	**M1**	**M2**	**M3**
Result 19	111788	16168	16825	40196	8527
Result 20	109229	15730	16451	32368	22293
Result 21	102118	14868	16809	35236	16367
**Mean**	107711.7	15588.7	16695.0	35933.3	15729.0
**STD**	4090.96	540.05	172.66	3233.58	5638.02
**%CV**	3.80	3.46	1.03	9.00	35.84
**Mean of all**	77757.1	23379.8	15784.2	29436.3	18375.8
**STD of all**	33855.33	13533.57	7486.75	10493.76	7750.81
**%CV of all**	43.54	57.89	47.43	35.65	42.18
	**%CV intra**	**9.62**			
	**%CV inter**	**8.69**			

MPC: maximal plasma concentration, CTRL: control> M1-3: coded drugs doses included in the drug combination

Supplementary Table S6. Calculation of Intra- and Interplate deviations of cell metabolic activity of SW620 3D co-cultures with tumor:fibroblast % 50:50.

	SW620 3D co-culturesmetabolic activity (raw data)				
	tumor:fibroblast 50:50				
**Exp1**	**Ctrl**	**MPC**	**M1**	**M2**	**M3**
Result 1	71584	48007	36119	50895	28887
Result 2	57301	51565	31387	51218	35966
Result 3	71448	43846	35531	47784	36611
**Mean**	66777.7	47806.0	34345.7	49965.7	33821.3
**STD**	6701.25	3154.47	2105.82	1548.30	3499.02
**%CV**	10.04	6.60	6.13	3.10	10.35
**Exp2**	**Ctrl**	**MPC**	**M1**	**M2**	**M3**
Result 4	75646	51820	26888	50537	37370
Result 5	72034	40072	27224	50623	27985
Result 6	69914	52443	27077	43690	40055
**Mean**	72531.3	48111.7	27063.0	48283.3	35136.7
**STD**	2366.36	5690.59	137.53	3248.17	5174.43
**%CV**	3.26	11.83	0.51	6.73	14.73
**Exp3**	**Ctrl**	**MPC**	**M1**	**M2**	**M3**
Resul 7	64573	28869	27741	42846	27661
Resul 8	58940	42309	20623	41837	20057
Resul 9	82636	31389	18857	40653	26930
**Mean**	68716.3	34189.0	22407.0	41778.7	24882.7
**STD**	10107.77	5833.15	3840.00	896.24	3425.29
**%CV**	14.71	17.06	17.14	2.15	13.77
**Exp4**	**Ctrl**	**MPC**	**M1**	**M2**	**M3**
Result 10	76875	55144	40106	54938	40661
Result 11	75987	52761	36137	51376	40364
Result 12	73360	29248	21815	51469	36041
**Mean**	75407.3	45717.7	32686.0	52594.3	39022.0
**STD**	1492.38	11686.38	7855.88	1657.66	2111.37
**%CV**	1.98	25.56	24.03	3.15	5.41
**Exp5**	**Ctrl**	**MPC**	**M1**	**M2**	**M3**
Result 13	71584	28887	56240	40578	45057
Result 14	57301	35966	52511	40009	50783
Result 15	71448	36611	56446	39877	47667
**Mean**	66777.7	33821.3	55065.7	40154.7	47835.7
**STD**	6701.25	3499.02	1808.38	304.15	2340.67
**%CV**	10.04	10.35	3.28	0.76	4.89
Exp6	**Ctrl**	**MPC**	**M1**	**M2**	**M3**
Result 16	64894	36559	52045	44501	35835
Result 17	65043.5	24512	51588	46592	48710
Result 18	65193	31608	45258	45359	48605
**Mean**	65043.5	30893.0	49630.3	45484.0	44383.3
**STD**	122.07	4944.09	3097.33	858.21	6044.74
**%CV**	0.19	16.00	6.24	1.89	13.62
**Exp7**	**Ctrl**	**MPC**	**M1**	**M2**	**M3**
Result 19	113259	36714	59128	43165	23558
Result 20	129998	34123	59246	44662	35700
Result 21	134262	33567	52786	35148	34005
**Mean**	125839.7	34801.3	57053.3	40991.7	31087.7
**STD**	9064.59	1371.38	3017.84	4177.05	5369.06
**%CV**	7.20	3.94	5.29	10.19	17.27
**Mean of all**	77299.1	39334.3	39750.1	45607.5	36595.6
**STD of all**	29802.63	18387.12	17001.07	20321.82	15125.71
**%CV of all**	38.55	46.75	42.77	44.56	41.33
	**%CV intra**	**8.84**			
	**%CV inter**	**6.70**			

MPC: maximal plasma concentration, CTRL: control> M1-3: coded drugs doses included in the drug combination

Supplementary Table S7. Calculation of Intra- and Interplate deviations of cell metabolic activity of DLD1 3D co-cultures with tumor:fibroblast % 50:50.

	DLD1 3D co-cultures metabolic activity (raw data)				
	tumor:fibroblast 50:50				
**Exp1**	**Ctrl**	**MPC**	**M1**	**M2**	**M3**
Result 1	43954	12262	21244	22975	13491
Result 2	42079	11455	20783	17310	14942
Result 3	52896	14341	21333	17783	15232
**Mean**	46309.7	12686.0	21120.0	19356.0	14555.0
**STD**	4719.73	1215.75	241.05	2566.29	761.62
**%CV**	10.19	9.58	1.14	13.26	5.23
**Exp2**	**Ctrl**	**MPC**	**M1**	**M2**	**M3**
Result 4	56814	22862	26706	24210	22518
Result 5	55676	23185	26338	26185	13121
Result 6	52681	23229	31491	21015	20074
**Mean**	55057.0	23092.0	28178.3	23803.3	18571.0
**STD**	1743.14	163.62	2347.22	2130.14	3980.80
**%CV**	3.17	0.71	8.33	8.95	21.44
**Exp3**	**Ctrl**	**MPC**	**M1**	**M2**	**M3**
Resul 7	49190	13626	19933	14470	12245
Resul 8	37287	14133	19483	16410	12009
Resul 9	31019	15012	16926	15605	-
**Mean**	39165.3	14257.0	18780.7	15495.0	12127.0
**STD**	7536.24	572.59	1324.25	795.81	118.00
**%CV**	19.24	4.02	7.05	5.14	0.97
**Exp4**	**Ctrl**	**MPC**	**M1**	**M2**	**M3**
Result 10	42628	17496	19145	17757	17348
Result 11	53709	19050	24006	21817	21725
Result 12	51255	20484	19976	23602	25014
**Mean**	49197.3	19010.0	21042.3	21058.7	21362.3
**STD**	4752.03	1220.17	2122.91	2445.72	3140.12
**%CV**	9.66	6.42	10.09	11.61	14.70
**Exp5**	**Ctrl**	**MPC**	**M1**	**M2**	**M3**
Result 13	36487	16076	25585	25228	26097
Result 14	35983	16763	26978	19732	24418
Result 15	45908	16706	27625	18265.0	22590.0
**Mean**	39459.3	16515.0	26729.3	21075.0	24368.3
**STD**	4564.54	311.29	851.19	2997.06	1432.16
**%CV**	11.57	1.88	3.18	14.22	5.88
**Exp6**	**Ctrl**	**MPC**	**M1**	**M2**	**M3**
Result 16	53808	6042	17673	20284	29014
Result 17	53620	7899	14691	15075	29963
Result 18	60135	8574	17528	21483	28547
**Mean**	55854.3	7505.0	16630.7	18947.3	29174.7
**STD**	3027.86	1070.57	1372.83	2781.56	589.14
**%CV**	5.42	14.26	8.25	14.68	2.02
**Exp7**					
Result 19					
Result 20					
Result 21					
**Mean**					
**STD**					
**%CV**					
**Mean of all**	47507.2	15510.8	22080.2	19955.9	20026.4
**STD of all**	16876.74	6854.43	8771.96	6939.42	7710.49
**%CV of all**	35.52	44.19	39.73	34.77	38.50
	**%CV intra**	**8.41**			
	**%CV inter**	**9.87**			

MPC: maximal plasma concentration, CTRL: control> M1-3: coded drugs doses included in the drug combination

Supplementary Table S8. Calculation of Intra- and Interplate deviations of cell metabolic activity of HCT116 3D co-cultures with tumor:fibroblast % 30:70 and 70:30.

HCT116 3D co-cultures cell metabolic activity (raw data)												
tumor:fibroblast 30:70							tumor:fibroblast 70:30					
**Exp1**	**Ctrl**	**MPC**	**M1**	**M2**	**M3**		**Exp1**	**Ctrl**	**MPC**	**M1**	**M2**	**M3**
Result 1	14692	4029	11983	11385	12448		Result 1	45682	7164	24870	19181	32820
Result 2	16204	3966	11599	11426	16889		Result 2	38533	7402	23572	16220	37978
Result 3	18188	4020	11483	9963	12688		Result 3	33504	8380	23851	19907	33112
**Mean**	16361.3	4005.0	11688.3	10924.7	14008.3		**Mean**	39239.7	7648.7	24097.7	18436.0	34636.7
**STD**	1431.57	27.82	213.67	680.21	2039.29		**STD**	4996.70	526.18	557.87	1594.73	2365.68
**%CV**	8.75	0.69	1.83	6.23	14.56		**%CV**	12.73	6.88	2.32	8.65	6.83
**Exp2**	**Ctrl**	**MPC**	**M1**	**M2**	**M3**		**Exp2**	**Ctrl**	**MPC**	**M1**	**M2**	**M3**
Result 4	36966	5141	17500	13425			Result 4	52942	7733	25179	18419	34989
Result 5	48808	5740	23237	14296	32094		Result 5	54944	9765	36233	15675	36447
Result 6	49843	7640	28453	15390	38564		Result 6	44599	9711	31565	18942	41601
**Mean**	45205.7	6173.7	23063.3	14370.3	35329.0		**Mean**	50828.3	9069.7	30992.3	17678.7	37679.0
**STD**	5841.63	1065.30	4473.23	803.93	3235.00		**STD**	4479.99	945.42	4530.91	1432.80	2836.43
**%CV**	12.92	17.26	19.40	5.59	9.16		**%CV**	8.81	10.42	14.62	8.10	7.53
**Exp3**	**Ctrl**	**MPC**	**M1**	**M2**	**M3**		**Exp3**	**Ctrl**	**MPC**	**M1**	**M2**	**M3**
Resul 7	23303	4324	15146	15833	24807		Resul 7	43996	9387	22825	18493	31639
Resul 8	25140	5357	13285	13364	19992		Resul 8	43538	11976	29870	23719	32574
Resul 9	24827	4302	15057	15677	23897		Resul 9	40966	8305	25716	22632	36086
**Mean**	24423.3	4661.0	14496.0	14958.0	22898.7		**Mean**	42833.3	9889.3	26137.0	21614.7	33433.0
**STD**	802.43	492.23	857.08	1128.93	2088.63		**STD**	1333.58	1540.20	2891.47	2251.52	1914.40
**%CV**	3.29	10.56	5.91	7.55	9.12		**%CV**	3.11	15.57	11.06	10.42	5.73
**Exp4**	**Ctrl**	**MPC**	**M1**	**M2**	**M3**		**Exp4**	**Ctrl**	**MPC**	**M1**	**M2**	**M3**
Result 10	23237	4484	8827	12411	19764		Result 10	44667	7273	18075	16850	26658
Result 11	30514	4359	8718	11681	16527		Result 11	49893	7880	21840	19240	33323
Result 12	16429	3900	10711	13320	19141		Result 12	40931	7513	17391	16759	26513
**Mean**	23393.3	4247.7	9418.7	12470.7	18477.3		**Mean**	45163.7	7555.3	19102.0	17616.3	28831.3
**STD**	5751.24	251.08	914.90	670.45	1402.35		**STD**	3675.54	249.61	1956.09	1148.71	3176.64
**%CV**	24.58	5.91	9.71	5.38	7.59		**%CV**	8.14	3.30	10.24	6.52	11.02
**CV intra**	**9.30**						**%CV intra**	**8.60**				
**CV inter**	**12.39**						**%CV inter**	**8.20**				

MPC: maximal plasma concentration, CTRL: control> M1-3: coded drugs doses included in the drug combination

Supplementary Table S9. Calculation of Intra- and Interplate deviations of spheroid size on day 2 of HCT116, SW620 and DLD1 3D co-cultures with tumor:fibroblast % 50:50.

	6 3D co-cultures growth kinetics day 2 (raw data)				
	tumor:fibroblast 50:50				
Exp1	size measurments/spheroid/well				
	315.9	304.6	317.6	304.3	287.2
	306.1	342.9	316.5	322.5	331.8
	305.3	301.2	342.2	333.7	307.7
	332.1	342.3	387.1	300.1	330.2
**Mean**	321.6				
**STD**	21.74				
**%CV**	6.76				
Exp2	size measurments/spheroid/well				
	321.3	333.4	318.1	349.1	328.8
	313.2	335.3	-	311.4	373.1
	315.9	346.1	308.2	343.2	335.5
	313.8	311.4	316.3	320	316.5
**Mean**	326.9				
**STD**	16.45				
**%CV**	5.03				
**Exp3**	size measurments/spheroid/well				
	335.9	377.9	347.2	347.9	337.1
	344.7	393.3	357.3	346.3	379
	351.4	336	357.8	356.1	351.7
	328.6	373.8	348.6	390.4	331.6
**Mean**	354.6				
**STD**	18.49				
**%CV**	5.21				
	**Mean of all**	**334.5**			
	**STD of all**	**24.00**			
	**%CV of all**	**7.18**			

	) 3D co-cultures growth kinetics day 2 (raw data)				
	tumor:fibroblast 50:50				
Exp1	size measurments/spheroid/well				
	310.4	290.1	300.8	379.7	361.5
	248.5	319.7	282.4	298.8	318.5
	340.6	320.6	288	331.7	299.1
	364.6	350	327.5	-	324.9
**Mean**	318.8				
**STD**	31.27				
**%CV**	9.81				
Exp2	size measurments/spheroid/well				
	351.7	333.8	338.4	341.8	-
	369.4	277.5	323.6	377.2	386.4
	374.1	370	365.3	328.8	327.1
	368.3	368.3	361.1	362.1	355.5
**Mean**	351.6				
**STD**	25.10				
**%CV**	7.14				
**Exp3**	size measurments/spheroid/well				
	244.3	272.6	265.6	287.6	278.4
	264	277.1	253.6	278.3	267.3
	238.7	268.9	269.5	314.1	259.4
	266.1	274.3	283.8	305.7	277.7
**Mean**	272.4				
**STD**	17.30				
**%CV**	6.35				
	**Mean of all**	**313.5**			
	**STD of all**	**41.21**			
	**%CV of all**	**13.15**			

	3D co-cultures growth kinetics day 2 (raw data)				
	tumor:fibroblast 50:50				
Exp1	size measurments/spheroid/well				
	306.8	329.7	291.9	319.2	322.1
	320.5	312.1	327.7	329.7	319.6
	-	306.2	307	310.1	296.1
	323.5	302.9	303.8	311.9	324.9
**Mean**	314.0				
**STD**	10.95				
**%CV**	3.49				
Exp2	size measurments/spheroid/well				
	237	-	229.7	252.1	248.3
	248.6	241.3	240.4	239.5	239.4
	245.8	225.5	236.7	261.8	250.3
	247.7	237.7	232.5	239	244.4
**Mean**	242.0				
**STD**	8.30				
**%CV**	3.43				
**Exp3**	size measurments/spheroid/well				
	366.4	344.7	302.4	305	290.9
	335.3	338.3	298.6	312.4	299.8
	309.9	260.8	317	262	312.5
	305.2	304.3	328.5	308	345
**Mean**	312.4				
**STD**	25.31				
**%CV**	8.10				
	**Mean of all**	**289.8**			
	**STD of all**	**37.40**			
	**%CV of all**	**12.90**			

Supplementary Table S10. Calculation of Intra- and Interplate deviations of spheroid size on day 5 of HCT116 and SW620 3D co-cultures with tumor:fibroblast % 50:50.

	T116 3D co-cultures growth kinetics day 5 (raw)					/620 3D co-cultures growth kinetics day 5 (raw)				
	tumor:fibroblast 50:50					tumor:fibroblast 50:50				
**Exp1**	**Ctrl**	**MPC**	**M1**	**M2**	**M3**	**Ctrl**	**MPC**	**M1**	**M2**	**M3**
Result 1	634.9	436.3	568.2	631	523.1	449.1	369	456.4	489.8	476.9
Result 2	550.3	434.6	506.3	643.6	577.6	444.4	366.3	445.3	460.1	431.6
Result 3	556.5	404	532.1	577.4	556	441	368.4	432.6	434.5	448.1
**Mean**	580.57	424.97	535.53	617.33	552.23	444.83	367.90	444.77	461.47	452.20
**STD**	38.50	14.84	25.39	28.70	22.41	3.32	1.16	9.72	22.60	18.72
**%CV**	6.63	3.49	4.74	4.65	4.06	0.75	0.31	2.19	4.90	4.14
**Exp2**										
Result 4	603.4	368	502.9	573.6	575.8	425.3	419.7	410	428.6	387.3
Result 5	581.8	393.6	482.9	580.9	533.7	441.6	353.4	393.1	422.2	402.9
Result 6	601.5	395.5	506.2	544.4	558.6	425.8	362.2	399.3	409	446.6
**Mean**	595.57	385.70	497.33	566.30	556.03	430.90	378.43	400.80	419.93	412.27
**STD**	9.77	12.54	10.29	15.77	17.28	7.57	29.40	6.98	8.16	25.10
**%CV**	1.64	3.25	2.07	2.78	3.11	1.76	7.77	1.74	1.94	6.09
**Exp3**										
Resul 7	588.7	403.6	458.3	530.4	523.5	-	373.9	435.7	-	443.9
Resul 8	584.2	374.4	421.5	545.6	517.5	512.6	358.5	425.1	465.5	457.1
Resul 9	551.8	336	475.3	548.3	539.4	474.6	356.5	433.6	480.1	458.7
**Mean**	574.90	371.33	451.70	541.43	526.80	493.60	362.97	431.47	472.80	453.23
**STD**	16.44	27.68	22.45	7.88	9.24	19.00	7.77	4.58	7.30	6.63
**%CV**	2.86	7.45	4.97	1.46	1.75	3.85	2.14	1.06	1.54	1.46
	**CV% intra**	**3.66**				**CV% intra**	**2.78**			
	**CV% inter**	**9.22**				**CV% inter**	**3.79**			

Supplementary Movies

Supplementary Movie S1. Kinetic movie of HCT116 3D co-culture spheroid formation

Representative movie of a HCT116 3D co-culture seeded at 1000 cells/well recorded for 24 hours. Culture consists of tumor cells (blue), CCD18co healthy colon fibroblasts (green) in ratio 1:1 and 5% endothelial ECRF24 cells (red). A discontinuous kinetic procedure with imaging every 30 minutes was performed fo r 24 hours. The movies are presented with 10 images/second.
Scalebar represents 200 µm.

Supplementary Movie S2. Kinetic movie of SW620 3D co-culture spheroid formation

Representative movie of a SW620 3D co-culture seeded at 1000 cells/well recorded for 24 hours. Culture consists of tumor cells (blue), CCD18co healthy colon fibroblasts (green) in ratio 1:1 and 5% endothelial ECRF24 cells (red). A discontinuous kinetic procedure with imaging every 30 minutes was performed for 24 hours. The movies are presented with 10 images/second. Scalebar represents 200 µm.

Supplementary Movie S3. Kinetic movie of DLD1 3D co-culture spheroid formation

Representative movie of a DLD1 3D co-culture seeded at 1000 cells/well recorded for 24 hours. Culture consists of tumor cells (blue), CCD18co healthy colon fibroblasts (green) in ratio 1:1 and 5% endothelial ECRF24 cells (red). A discontinuous kinetic procedure with imaging every 30 minutes was performed for 24 hours. The movies are presented with 10 images/second. Scalebar represents 200 μm.
